# HLAreporter: a tool for HLA typing from next generation sequencing data

**DOI:** 10.1186/s13073-015-0145-3

**Published:** 2015-03-16

**Authors:** Yazhi Huang, Jing Yang, Dingge Ying, Yan Zhang, Vorasuk Shotelersuk, Nattiya Hirankarn, Pak Chung Sham, Yu Lung Lau, Wanling Yang

**Affiliations:** Department of Paediatrics and Adolescent Medicine, Li Ka Shing Faculty of Medicine, The University of Hong Kong, 21 Sassoon Road, Hong Kong, Hong Kong; Department of Psychiatry, Li Ka Shing Faculty of Medicine, The University of Hong Kong, 21 Sassoon Road, Hong Kong, Hong Kong; Department of Pediatrics, King Chulalongkorn Memorial Hospital, Faculty of Medicine, Chulalongkorn University, Bangkok, Thailand; Immunology Unit, Department of Microbiology, Faculty of Medicine, Chulalongkorn University, Bangkok, Thailand

## Abstract

Human leukocyte antigen (HLA) typing from next generation sequencing (NGS) data has the potential for widespread applications. Here we introduce a novel tool (HLAreporter) for HLA typing from NGS data based on read-mapping using a comprehensive reference panel containing all known HLA alleles, followed by *de novo* assembly of the gene-specific short reads. Accurate HLA typing at high-digit resolution was achieved when it was tested on publicly available NGS data, outperforming other newly developed tools such as HLAminer and PHLAT. HLAreporter can be downloaded from http://paed.hku.hk/genome/.

**Electronic supplementary material:**

The online version of this article (doi:10.1186/s13073-015-0145-3) contains supplementary material, which is available to authorized users.

## Background

The human leukocyte antigens (HLAs) include a large number of genes crucial to immune system function. They play important roles in immune responses to infection, transplant rejection, pathogenesis of autoimmune diseases, adverse drug reaction, and cancer development. Thus, HLA typing is very important for both clinical laboratories and biomedical research. For example, HLA is highly associated with many complex diseases such as autoimmune disease and cancer and typing HLAs from next-generation sequencing (NGS) data can have widespread application in identifying the associated genes for complex diseases. HLA is also the key for many adverse drug responses and transplant rejection. Thus, typing HLA from NGS data can at least serve as a preliminary population screening tool to identify individuals who might have potential adverse drug responses or are potential organ donors, although exact clinical use would require more stringent standards and procedures. Since having NGS data for large numbers of healthy individuals is rapidly becoming a reality, the potential benefits of HLA screening using this type of data is multi-fold.

However, HLA typing has always been challenging due to the complexity of this group of genes, including the existence of large number of alleles for most HLA genes, major sequence difference between these alleles, sequence similarity among the paralogous HLA genes, and long range linkage disequilibrium in this region [1,2]. For example, for the HLA-DRB1 gene alone, over a thousand alleles have been reported in human populations according to the IMGT/HLA database (IMGT/HLA 2012, release 3.10.0) [3]. In addition, many HLA-DRB5 alleles have great sequence similarity to those of DRB1, adding more difficulties for accurately calling HLA alleles from sequencing data [4].

HLA typing has been done via various technologies, such as serological, cellular, and molecular assays [5]. Sequencing-based methods have been rapidly gaining popularity due to technology advancement, especially in research settings. With the development of NGS, large amounts of sequencing data are becoming widely available. Although most the data were not generated for this purpose, they still provide valuable resources for HLA typing. NGS data might be useful in multiple contexts, such as preliminary screening for potential organ donors or for individuals that are potentially susceptible to adverse drug responses, for risk prediction for complex diseases, and population genetic studies [6,7]. They also provide much more comprehensive information on this region than any other traditional methods of HLA typing, potentially useful in sorting out the complex structure of the genetic variants in this region.

However, due to the complexity of the HLA loci, the large amount of NGS data has not yet been rendered informative for HLA genotypes. Many HLA typing efforts have been made by mining NGS data, including the alignment-based method that relies on counting the number of short reads aligned to each specific allele [8], the assembly- and scoring-based method that takes into account good quality contigs and their scores for each candidate HLA allele [9]. These methods capitalize on the increasing accessibility and affordability of NGS sequencing and have greatly reduced the time and cost required to make an HLA call compared with traditional standard PCR-based solutions. Unfortunately, all these methods are only capable of achieving low-digit resolution and perform poorly at higher-digit resolution, which is required for clinical applications.

In this study, we introduce a novel approach for accurate HLA typing at high-digit resolution based on a strategy of comparing sequence reads with a comprehensive reference panel containing all the known HLA alleles for high efficiency mapping, followed by assembly of the mapped reads to contigs, stepwise matching and designation of the contigs to HLA alleles and decision on HLA allele calling. Testing of the method on a set of public and internal whole exome sequencing (WES) data demonstrated that this new method is capable of reporting HLA alleles at high-digit resolution with great accuracy. We also conducted a preliminary analysis of WES data from 149 NGS samples generated in-house. HLA calling results demonstrated consistent allele frequencies to those recorded in the Allele Frequency Net Database (AFND) [10] of the same population. Certain long range HLA haplotypes across class I and II genes reported in AFND were also observed in our dataset. These preliminary results highlight the potential applications of this method for HLA calling from NGS data, which may have significant implications in many important clinical contexts.

## Methods

### Classification of short sequencing reads to specific HLA genes through mapping using a comprehensive reference panel

In order to achieve accurate HLA typing, the first essential step is to accurately classify the short sequencing reads with regard to the specific HLA genes they are derived from. Many of the short sequencing reads from HLA genes are not mapped properly or are labeled as unmapped for most NGS data processing procedures due to great allelic differences and sequence similarity between paralogous genes. Recognizing this, we designed a comprehensive reference panel (CRP) for classifying reads according to their corresponding HLA genes. Allele differences were fully accounted for during mapping by adopting all the known HLA alleles in the IMGT/HLA database as references, which ensured complete capture of the HLA reads for further analysis and accurate classification. Mapping was performed using Burrows-Wheeler Aligner (BWA) v.0.6.1 [11] with default parameters, using all the raw reads from fastq file for NGS data against all the reference sequences in the CRP. In this work, we mainly considered a gene’s polymorphic exons for HLA typing (exons 2, 3, and 4 for class I genes HLA-A, HLA-B, and HLA-C, and exons 2 and 3 for the class II genes). In order to capture reads containing partial intron sequences, a comprehensive panel of references was designed by appending 50 bp of intron sequences extracted from the IMGT/HLA database to sequences from both ends of a reference exon. For the alleles without intron sequence information in the IMGT/HLA database, corresponding intron sequences from other alleles of the same gene were used to include intron sequences at the junctions. As a result, the panel is capable of capturing short reads partly falling outside of the targeted exons by as many as 50 bp, for mapping from both WES and whole genome sequencing (WGS) data.

**Figure 1:**
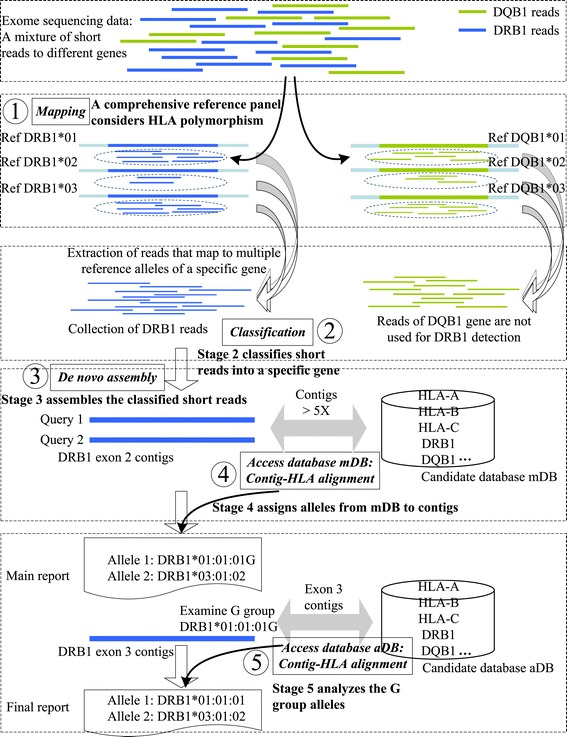
**HLAreporter detection flow using the HLA-DRB1 gene as an example.** Classification of reads to a specific gene using CRP panel-based mapping is shown in stages 1 and 2. Assembly and contig-HLA matching are shown in stages 3, 4, and 5.

In addition to the targeted genes that we aimed to type (that is, HLA-A, −B, −C, −DRB1, −DQB1, −DQA1, −DPB1, −DRB3, 4, 5), we also included all known allelic sequences of a set of minor HLA genes (that is, HLA-E, −F, −G, −H, −J, −K, −L, −V, −P, −DMA, −DMB, −DOA, −DOB, −DPA1, −DRA) in the CRP panel for mapping accuracy. These genes serve as ‘mapping competitors’ to ensure accurate mapping of the sequencing reads. After classification, short reads mapped to a specific gene were collected in order to assemble them into contigs for further analysis (Figure 1).

We excluded those ambiguous reads where a short read mapped to a gene from the mapping panel could also be perfectly mapped to another gene. Reads with equal mapping score towards multiple genes but with imperfect sequence alignment were retained for further analysis since this level of similarity is expected among different HLA genes. The short reads mapped to a particular HLA gene were assembled respectively using *de novo* assembly. An assembler called TASR [9] was used here for the *de novo* assembly (for a detailed description of the TASR algorithm see Warren *et al*. [9]). During this process, only reads with a 100% match in the overlapped region were assembled. On average, 30% of short reads with mismatches would have to be excluded based on the NGS data used in this study, effectively eliminating potential effects of sequencing error on assembly.

### Design of reference database of HLA alleles for matching assembled contigs

We designed two reference databases of HLA alleles for matching the assembled contigs to the corresponding HLA alleles, one with sequences for the major polymorphic exons (exons 2 and 3 for class I genes and exon 2 for class II genes) and one with additional sequences for the minor polymorphic exons (exon 4 for class I genes and exon 3 for the class II genes). The first reference database designed was the major database (mDB), which contains sequence information of all the known alleles on the major polymorphic exons from the IMGT/HLA database, and this database is queried first using each assembled contig (exons 2 and 3 for class I genes and exon 2 for class II genes). When multiple HLA alleles have identical nucleotide sequences across the major exons, an upper case ‘G’ is appended to their names as a suffix based on HLA nomenclature [12] and these alleles are further examined. Unlike the CRP, which was designed for mapping of the sequencing reads that may contain intron sequences, the reference database here does not contain intron extensions, and no ‘competitor sequences’ are included.

The second reference database is designated the additional database (aDB), which records the minor polymorphic exon sequences for all the ‘G’ group alleles. When a specific allele cannot be designated via mDB, the contigs corresponding to minor exon sequences are examined by aligning them to the candidate alleles in aDB. We separated the minor exons from the major ones since a number of alleles in the IMGT/HLA database do not have information on the minor exons. This design also enhances the efficiency of the analysis on the assembled contigs.

### A stepwise HLA typing process

To assign candidate alleles to the assembled contigs, the targeted contigs are then matched to the sequences in the two databases, mDB and aDB, sequentially. To guarantee accuracy, a stringent standard is adopted during the contig-HLA allele matching process, where only a perfect match to the candidate alleles on the exonic regions is considered. Contigs supported by an average read coverage depth of less than five-fold are also excluded since contigs with lower depth might be less reliable. All contigs with different lengths supported by an average depth of five-fold or above are considered in cases where not enough long contigs could be generated by the assembler for certain HLA alleles. By analyzing all the assembled contigs using a scoring system based on the length of the contig in the exonic region and the coverage depth, the candidate HLA alleles that match those assembled contigs are assigned (for the scoring system and the assignment algorithm see Warren *et al*. [9]). Briefly, the score of a contig is the product of the contig size (base pairs), the average coverage depth of the contig, and the percentage of the contig’s exonic sequence. Accordingly, the score of an allele supported by multiple contigs (for example, exons 2 and 3 for class I genes) is the sum of the contig scores. Each candidate allele is measured by the corresponding contig scores and is sorted in a descending order. Based on the sorted scores, HLA alleles are reported whenever a uniquely matched contig that has not already been assigned is detected. The overall flow of this technique is presented in Figure 1.

Since we only allowed for perfect matches between assembled contigs and the HLA alleles in the database, any assembled contigs with mismatches were not considered at this step. For the purpose of novel allele detection, HLAreporter would document all the assembled contigs for the main exons, and report these contigs and their quality to users so that further analysis of them could reveal novel alleles. Similarly, sample contamination may introduce a third or fourth HLA allele for a given gene and this will also be documented for the user’s attention.

### Application of HLAreporter on whole exome sequencing data

Data from 82 samples with a total of 791 verified HLA alleles using experimental methods were selected to test the performance of HLAreporter, including 62 publicly available samples [1,13-15] and 20 internal samples from the Thai population (downloadable from [16]). WES data in fastq format were downloaded from the 1000 Genomes Project [17] and the HapMap Project [18]. Of the 62 public samples, the 11 samples from 1000 Genomes Project were generated using Illumina GAIIx and allelic HLA typing was performed using SeCore HLA Sequencing Reagents (Life Technologies Corporation, Grand Island, New York, USA). The 51 HapMap samples were released in 2013 by the Baylor College of Medicine (BCM) and Washington University Genome Sequencing Center (WUGSC). We selected these samples because they have publicly available calls on HLA class I and class II genes and relatively longer read length and higher coverage depth, a feature of the more recently released samples. The details of the WES data, including 62 publicly available samples with 711 verified HLA types, can be found in Table S1 in Additional file 1.

A preliminary analysis of in-house WES data on the Hong Kong population was also conducted using HLAreporter. The raw data in fastq format were generated using Illumina HiSeq 1500 with a read length ranging from 90 to 101 bases. A total of 149 Hong Kong Chinese samples were included, each of which is an independent founder individual. There is no known association between the conditions of these individuals and HLA alleles and the details of these 149 samples can be found in Table S1 in Additional file 1. The WES data are accessible through our website.

Using NGS data for HLA typing, there could be phasing ambiguities. For those HLA genes with phasing issues, alleles with higher frequency in the public database were assigned over those with lower population frequencies. For example, allele pairs (A*02:03:01G; A*31:01:02G) and (A*02:152; A*unknown-allele) could both explain the observed genotypes for an individual due to phasing ambiguity. Since A*02:152 has a much lower frequency than A*02:03:01G and there is also an unknown allele with 0 reported population frequency in the IMGT/HLA database, allele pair (A*02:03:01G; A*31:01:02G) would be assigned here.

We used PHASE version 2 [19] to predict long range HLA haplotypes. PHASE is a widely used tool that makes use of Bayesian methods for haplotype reconstruction and recombination rate estimation from population data. PHASE supports SNPs as input; therefore, we treated each HLA allele as if it is a SNP allele to run PHASE. The top 50 alleles for each HLA gene based on their population allele frequency were included as index alleles for phasing as there is an allele number limit allowed for PHASE. Then we used these indexes to represent HLA alleles and ran multi-allelic phasing to obtain the long range haplotypes.

This study is conducted in compliance with the Helsinki Declaration (Edinburgh, October 2000) and in accordance with local legislation. The research is approved by the Institutional Review Board of the University of Hong Kong and Hospital Authority Hong Kong West Cluster, as well as the Institutional Review Board of Chulalongkorn University Faculty of Medicine, Bangkok, Thailand. All participants gave informed consent to take part in the study.

## Results

### Mapping efficiency

In this method, a CRP was used for short read collection for HLA genes. To study the mapping efficiency for this group of genes, we compared the number of reads captured for the HLA-DRB1 gene using a traditional single reference-based mapping method and the CRP-based mapping developed in this study, using BWA as the mapping tool in both cases.

**Figure 2:**
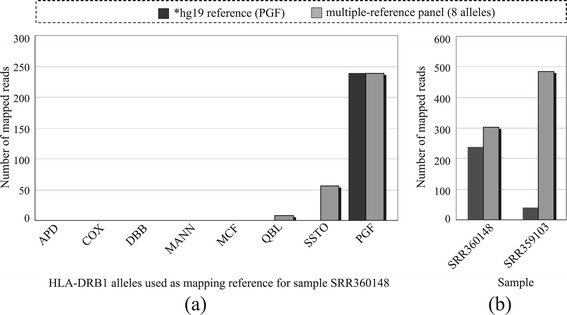
**Mapping efficiency for HLA-DRB1 genes.** Sample SRR360148 is heterozygote with alleles DRB1*01:01 and DRB1*15:01. Sample SRR359103 is heterozygote with alleles DRB1*03:01 and DRB1*07:01. **(a)** Difference in the number of reads captured on the exon 2 region for sample SRR360148. Clearly a multiple allele-based mapping panel that contains eight different alleles of HLA-DRB1 outperforms a single reference hg19*15:01:01:01 (that is, PGF). **(b)** The total number of reads captured using hg19*15:01:01:01 as reference only versus a multiple allele-based reference panel for samples SRR360148 and SRR359103. Using one mapping reference would lose quite a number of short reads.

As can be seen in Figure 2a, compared with using single reference hg19 only during mapping, sample SRR360148, whose DRB1 alleles are *01:01 and *15:01, was more effectively mapped by a reference panel with multiple alleles corresponding to the eight haplotypes in hg38, namely APD, COX, DDB, MANN, MCF, QBL, SSTO, and PGF (the allele for hg19). The short reads matching the two DRB1 alleles *01:01 and *15:01 were better captured by the multiple reference panel, even though allele *01:01 was still underrepresented by the eight-haplotype panel and far fewer short reads were captured compared with those corresponding to allele PGF (*15:01). Clearly, using a single allele hg19 *15:01:01:01 as the reference can only capture those short reads similar to itself, which would result in a loss of short reads derived from allele *01:01 and an incorrect DRB1 designation.

Figure 2b summarizes the total number of reads captured for samples SRR360148 and SRR359103. Using a single allele as the mapping reference, as done by most WES processing tools, would lose quite a number of short reads and likely result in incorrect HLA typing. For SRR360148, the number of short reads that a single reference can capture amounts to only about 75% of that captured by the multiple allele reference panel. The differences were much greater for sample SRR359103, which has alleles DRB1*03:01 and *07:01 for this gene, where the majority of sequence reads were not captured using reference hg19 *15:01:01:01. This resulted in an HLA detection failure for this sample when we used those reads for HLA typing, emphasizing the deficiency of traditional mapping approaches for HLA detection.

### Predictions of HLA class II genes

Predictions of HLA class I and class II genes for the 11 publicly available 1000 Genomes samples with adequate NGS data are presented in Table 1 (a description of data quality is presented in Table S1 in Additional file 1). Results for 51 additional HapMap samples and 20 internal Thai samples are shown in Table S2 in Additional file 1. Predictions made by HLAminer [9] using *de novo* assembly are also shown as a comparison. The columns ‘HLA-DQB1(exon 2)’ and ‘HLA-DQB1 (exon 2&3)’ represent predictions made by examining exon 2 only and predictions after further examining exon 3, respectively.

**Table 1:** **HLA predictions of class I and class II genes**

	**HLAminer**	**HLAreporter**	**HLAminer**	**HLAreporter**	**HLAminer**	**HLAreporter**	**HLAminer**	**HLAreporter**	**DRB1**	**HLAminer**	**HLAreporter**	**DQB1**
**ID** ^**(SRR)a**^	**HLA-A**	**HLA-A**	**HLA-B**	**HLA-B**	**HLA-C**	**HLA-C**	**HLA-DRB1**	**DRB1(exon 2)**	**(exon 2&3)** ^**b**^	**HLA-DQB1**	**DQB1 (exon 2)**	**(exon 2&3)** ^**b**^
359102	*30:01; *30:02; *30:04; *66:01	*30:02:01G; *66:01:01G	*15:83; *18:01; *18:26; *41:01; *45:01; *50:01	*18:01:01G; *41:02:01	*05:01; *17:01	*05:01:01G; *17:01:01G	*03:01; *07:01	*03:01:01G	*03:01:01	*02:01	*02:01:01G	*02:01:01
359103	*01:01; *01:03; *02:01; *02:03; *11:02; *68:08	*01:01:01G; *02:01:01G	*18:01; *18:03; *57:01	*18:01:01G; *57:01:01G	*07:01	*07:01:01G	*03:01; *07:01	*03:01:01G; *07:01:01G	*03:01:01; *07:01:01	*02:01; *03:03	*02:01:01G; *03:03:02G	*02:01:01; *03:03:02
359108	-	*03:01:01G; *68:02:01G^p^	-	*35:01:01G; *53:01:01	*04:01	*04:01:01G	-	*04:05:01; *08:04:01	*04:05:01; *08:04:01	-	*03:01:01G; *03:02:01G	*03:01:04; *03:02:01
359298	*11:01; *11:02; *11:50; *24:02; *24:07; *24:20^c^	*11:02:01G; *24:07^p^	*27:04; *27:25; *39:34; *40:02; *40:06	*27:04:01G; *39:05:01	*08:01; *08:21; *12:02; *12:03	*08:01:01G; *12:02:01G^p^	*04:03; *08:03; *12:01; *14:54^c^	*08:03:02; *12:02:01	*08:03:02; *12:02:01	*03:01; *06:01	*03:01:01G; *06:01:01G	*03:01:01; *06:01:01
359295	*02:03; *03:01	*02:03:01G; *03:01:01G^p^	*35:01; *35:03; *37:01; *55:02; *55:48; *56:01	*35:03:01G; *55:02:01G^p^	*01:02; *04:01; *04:03; *12:03; *15:02; *15:16^c^	*04:01:01G; *12:03:01G	*04:03; *07:01; *08:03; *14:05	*08:02:01; *14:05:01	*08:02:01; *14:05:01	*03:02; *03:03; *03:05; *05:03	*04:02:01; *05:03:01G	*04:02:01; *05:03:01
360655	*30:01; *30:02; *30:04; *32:01; *74:01; *74:11	*30:02:01G; *74:01:01G	*15:03; *57:01; *57:06; *57:11	*15:03:01G; *57:03:01^p^	*02:02; *02:11; *07:01	*02:10; *07:01:01G^p^	*07:01; *08:03; *11:01; *13:02	*11:01:02; *13:02:01	*11:01:02; *13:02:01	*05:01; *05:03; *06:09	*05:02:01G; *06:09:01	*05:02:01; *06:09:01
360288	*02:01	*02:01:01G; *02:11:01G	*15:01; *15:07; *15:32; *35:14; *58:01	*15:04; *35:05:01	*01:02; *04:01; *04:03; *04:06	*01:02:01G; *04:01:01G	*04:03; *07:01	*04:11:01; *09:01:02	*04:11:01; *09:01:02	*03:02	*03:02:01G; *03:03:02G	*03:02:01; *03:03:02
360391	*02:01; *02:48; *68:01	*02:01:01G; *68:01:02G^p^	*07:02; *40:02; *40:06	*07:02:01G; *40:02:01G^p^	*03:03; *03:04; *07:02	*03:04:01G; *07:02:01G^p^	*01:01; *07:01	*01:03; *09:01:02	*01:03; *09:01:02	*03:03; *05:01	*03:03:02G; *05:01:01G	*03:03:02; *05:01:01
360148	*01:01; *02:01; *36:01	*02:01:01G; *36:01	*07:02; *35:01; *35:41; *40:01; *40:79; *53:01^c^	*35:01:01G; *40:01:01G^p^	*03:02; *03:04; *04:01; *04:03; *15:02; *15:17^c^	*03:04:01G; *04:01:01G	*01:01; *01:02; *07:01; *15:01	*01:01:01G; *15:01:01G	*01:01:01; *15:01:01	*05:01; *06:02	*06:02:01G; *05:01:01G	*06:02:01; *05:01:01
359301	*02:03; *11:02; *31:01; *32:01; *74:01; *74:11	*02:03:01G; *31:01:02G^p^	*13:01; *48:01	*13:01:01G; *48:01:01G	*03:03; *03:04	*03:03:01G; *03:04:01G	*07:01; *08:03; *11:01	*11:01:01G; *13:12:01	*11:01:01; *13:12:01	*03:01	*03:01:01G	*03:01:01
359098	-	*03:01:01G; *68:02:01G^p^	-	*35:01:01G; *53:01:01	*04:01	*04:01:01G	-	*04:05:01; *08:04:01	*04:05:01; *08:04:01	-	*03:01:01G; *03:02:01G	*03:01:04; *03:02:01

^a^‘SRR’ is the prefix of each sample name, which is not explicitly shown in the table due to space limitations. ^b^All alleles with identical exon 2 and 3 sequences are reported. For example, after examining exons 2 and 3 of allele DQB1*03:03:02G, HLAreporter reports alleles 03:03:02:01/03:03:02:02/03:03:02:03. Since the last two digits out of eight digit-based HLA nomenclature are determined by intronic sequences, we only present the first six digits *03:03:02 in the table after examining the minor exon. ^c^Additional ambiguity at four-digit resolution is not shown. ^p^Phase was reported.

As we can see from Table 1, for HLA class II genes, our results show complete consistency with the reported alleles at the four-digit resolution (the 110 known HLA types are presented in Table S1 in Additional file 1). In contrast, HLAminer mistyped heterozygosity to homozygosity in the case of SRR360288, failed to achieve four-digit resolution in the case of SRR360655, and reported an incorrect result at two-digit resolution in the case of SRR359295, just to give a few examples. It is observed that, for some class II alleles, examining exon 2 sequences alone would be sufficient (for example, for DQB1*06:09:01, polymorphism is not currently found in any other exons or introns except exon 2). While for the alleles with identical exon 2 sequences but differences in other exons, further examination of the exon 3 region would be necessary.

### Typing results on HLA class I genes

While accurate predictions without ambiguity were achieved for HLA class II genes in all the samples checked, HLA typing of class I alleles appeared to be more affected by phase ambiguity. Generally, phase becomes an issue when the size of the non-polymorphic gap between any two alleles is greater than the read length, since different combinations could result in different alleles. We would report this phase ambiguity to users so that further measures can be taken accordingly (Table S3 in Additional file 1). We adopted the same measures as in HLAminer by Warren *et al*. [9], that is, sensitivity, specificity, and ambiguity as assessment metrics. To summarize, for HLAminer, sensitivity, specificity, and ambiguity on this group of genes were 85%, 88%, and 56%, respectively. Of 66 class I alleles tested, 25 alleles (38%) were accurately reported by HLAminer without ambiguity. On the other hand, although with phase issues on ambiguous haplotypes, all predictions made by HLAreporter were consistent with the reported HLA alleles at the four-digit resolution.

### Prediction accuracy

Thus, the reliability of this tool was verified using the 110 HLA alleles from 1000 Genomes samples, whose typing was determined through standard PCR-based methods. In addition, the 51 HapMap samples and 20 internal samples from the Thai population were also tested. The HLA predictions were completely consistent with the experiment-based typing results for all the samples that passed our quality test. Table 2 presents the statistics for the HapMap samples with 601 known HLA alleles. With 20-fold coverage depth for 98% of the sequences in the exonic region, we achieved 100% typing accuracy at a four-digit resolution for all HLA genes (Table 2, row ‘10 × = 100% and 20× ≥98%’). When the quality standard was reduced to 90% of the exonic regions with coverage of 20-fold (Table 2, row ‘10 × = 100% and 20× ≥90%’), we still achieved respectable performance at the four-digit resolution, particularly for class II genes (accuracy > 99%). Although there were certain ambiguities at the four-digit resolution, at the two-digit resolution, accuracy remained at 100%. HLA-DQA1 apparently is the most tolerant gene to low data quality, demonstrating 100% accuracy at the four-digit resolution even when the quality standard was reduced to 95% of the regions with only 10-fold coverage depth (Table 2). This is probably due to the least polymorphic nature of this HLA gene. HLA-DRB1 and -DQB1 failed to achieve 100% accuracy at two-digit resolution under this lower coverage depth, where three genes were mistyped as homozygous since one of the two alleles was missed in each case (Table S2 in Additional file 1).

**Table 2:** **Statistics of HLA typing results from 51 HapMap samples**

**Quality standard (QS)** ^**a**^	**HLA gene**	**Total number of genes**	**Number of QS passes** ^**a**^	**Pass QS (%)** ^**a**^	**Four-digit (%)** ^**b**^	**Two-digit (%)** ^**b**^
10 × = 100% and 20× ≥98%	HLA-A	51	3	6%	100%	100%
	HLA-B	51	7	14%	100%	100%
	HLA-C	51	4	8%	100%	100%
	HLA-DRB1	46	28	61%	100%	100%
	HLA-DQB1	51	20	39%	100%	100%
	HLA-DQA1	51	20	39%	100%	100%
10 × = 100% and 20× ≥90%	HLA-A	51	18	35%	81%	100%
	HLA-B	51	18	35%	83%	100%
	HLA-C	51	11	22%	100%	100%
	HLA-DRB1	46	40	87%	99%	100%
	HLA-DQB1	51	27	53%	100%	100%
	HLA-DQA1	51	35	69%	100%	100%
10× ≥95%	HLA-DRB1	46	45	98%	96%	98%
	HLA-DQB1	51	46	90%	91%	99%
	HLA-DQA1	51	43	84%	100%	100%

^a^Quality standard (QS) ‘10 × ’ represents the percentage of locations with coverage depth greater than 10-fold on the targeted exon. Accordingly, ‘10× ≥95%’ means the pre-defined percentage (that is, ‘10 × ’) is 95% or above. (‘20 × ’ has a similar definition). ^b^The four-digit (two-digit) percentage is equal to the number of HLA calls at four-digit (two-digit) resolution without ambiguity divided by the total number of alleles.

To summarize, in total, data from 82 samples with 791 known HLA types were tested using HLAreporter. With a 20-fold coverage quality standard for most targeted exonic sequences (that is, row ‘10 × = 100% and 20× ≥98%’ in Table 2), all 288 alleles (36% of all the tested alleles) that passed the quality threshold were correctly typed at four-digit resolution. Using a more lenient quality standard for the less polymorphic class II genes (90% of the regions with coverage of 20-fold), of all the 370 alleles (47% of all the tested alleles), only one HLA-DRB1 allele was ambiguous at the four-digit resolution, while the other 369 alleles were correctly typed. Based on these results, for HLAreporter, calls made based on coverage lower than 20-fold in more than 2% of the exonic sequences will be accompanied with a warning sign for the user to check the quality of the call. Since NGS is becoming more and more accurate and with higher coverage depth, our algorithm is advantageous with its call accuracy despite its demand on data coverage and quality.

For the 149 samples whose data were produced in house, we did not have HLA allele information available. To further test the accuracy of HLA typing by HLAreporter, we randomly chose five samples and performed PCR amplification and Sanger sequencing on HLA-DRB1 exon 2. The HLA typing results show complete consistency with our predictions (result for sample PaedA51 is shown as an example in Figure S1 in Additional file 1).

During the assembly process, only reads with a 100% match in the overlapped region were assembled, eliminating potential effects of sequencing errors on assembly. In addition, all predictions were based on the assembled contigs that have perfect match with sequences in the IMGT/HLA database in the exonic regions. We also checked the quality of assembled contigs on the exon 2 region that supported the typing results of the HLA-DRB1 gene for three samples. We observed that most predictions achieved a coverage depth of 15-fold or above, suggesting reliable predictions. A balanced coverage of the two alleles in each case was also achieved (Table S4 in Additional file 1). Further, we examined the coverage of this region using the Integrative Genomics Viewer (IGV), a tool that allows viewing of the short reads mapped to the targeted exons. And the read patterns observed using the Integrative Genomics Viewer demonstrated consistency with the predicted results (Figure S2 in Additional file 1).

### HLA profiles detected in the Hong Kong Chinese population

**Figure 3:**
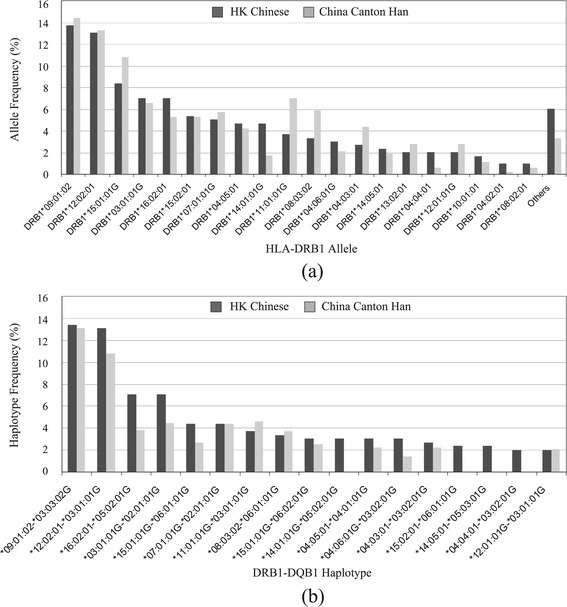
**HLA distribution profiles. (a)** Allele frequency distribution of HLA-DRB1 in the Hong Kong (HK) Chinese and China Canton Han populations. **(b)** Haplotype frequency distribution of DRB1-DQB1 in the Hong Kong Chinese and China Canton Han populations.

We used data from the 149 samples generated in-house to study the HLA profiles in the Hong Kong Chinese population. Distribution of HLA-DRB1 alleles is presented in Figure 3a and the distributions of other HLA genes are shown in Figure S3 in Additional file 1. Allele frequencies in a China Canton Han population with 264 individuals are also presented (derived from [20]). According to their descriptions, the HLA alleles were typed using the PCR sequence-specific oligonucleotide probe (SSOP) typing method. The database provides allele information with four-digit resolution (for example, DRB1*03:01), so we used the alleles with the same first four digits for comparison (for example, DRB1*03:01:01G versus DRB1*03:01 in the database). The top three DRB1 alleles with highest frequencies are *09:01:02, *12:02:01, and *15:01:01G. Indeed, these alleles are known to be common in Asian populations (for the AFND Canton Han population,*09:01, 14%; *12:02, 13%; *15:01, 10%). Compared with records in AFND on Europeans, some population differences in allele frequencies were observed, such as those for *12:02 and *12:01. While European populations have a higher frequency for *12:01, Hong Kong Chinese have a five times higher frequency for *12:02.

### From alleles to haplotypes

Figure 3b presents the prediction of major DRB1-DQB1 haplotypes calculated by PHASE. The haplotype distribution is also compared with that provided in the AFND database on matched populations. The major haplotypes DRB1*09:01:02-DQB1*03:03:02G and DRB1*12:02:01-DQB1*03:01:01G were clearly observed with the highest frequency in our data, consistent with the record in AFND.

Notably, strong linkage disequilibrium of the HLA genes across a long distance was observed. For example, the DRB1*09:01:02 and DQB1*03:03:02G alleles (Figure S3 in Additional file 1) both have an allele frequency of 13.5% in Hong Kong Chinese, while haplotype DRB1*09:01:02-DQB1*03:03:02G also presented with a frequency at about 13.5%, indicating that these two alleles have near absolute linkage disequilibrium with each other. Likewise, haplotype DRB1*12:02:01-DQB1*03:01:01G has nearly the same frequency as allele DRB1*12:02:01. Long range haplotypes might play an important role in disease association and drug response; thus, phasing these haplotypes for the HLA alleles is necessary in association studies.

## Discussion

We have shown that our approach is efficient for HLA typing from whole exome sequencing data. The reliability of this tool is verified by testing 791 known HLA class I and class II alleles from 82 samples, whose HLA alleles were experimentally confirmed. To achieve reliable predictions, a stringent assembly procedure was conducted to form contigs (zero mismatch tolerance), followed by a stringent HLA assignment process to assign alleles (zero mismatch tolerance on exonic regions), processes that would ensure accuracy for HLA calls.

Since the proposed technique relies on *de novo* assembly, read length is critical for typing accuracy. A shorter read length would worsen the phase issue. Generally, phase becomes an issue when the size of the non-polymorphic gap between any two alleles is greater than the read length (100 bp in our tested 1000 Genomes data), since different combinations might result in different alleles. In addition, phase also becomes an issue when sequences between two exons could have different combinations, which has been reported in the IMGT/HLA database. For example, for HLA alleles C*08:21, C*08:01:01G, C*08:16:01, C*12:02:01G, and C*12:49, there is a 110 bp gap within exon 2 and an inter-exon gap between exon 2 and exon 3, with different combinations specifying different alleles. Apparently this problem cannot be solved by the current sequencing technology using short reads and paired-end information of reads can only partially alleviate the problem. While class II genes seem to have little phase problem, class I gene typing is significantly affected by this issue.

To achieve high accuracy, data with good coverage on HLA genes are also essential. A depth test on each exon of the targeted gene is necessary to ensure accurate typing. If sequencing reads were poorly captured during the enrichment process on exonic fragments, it would be unlikely to properly detect HLA alleles. While there is no golden standard, 30-fold depth on every location of the targeted exons is recommended for adequate coverage and HLA calls (Figure S4 in Additional file 1). Balanced capture of the different alleles is also important, a process that should be considered during the design of the probes for enriching exonic genomic fragments. In the 82 real samples we tested, it was shown that 20-fold depth on every targeted location could reach 100% accuracy at four-digit resolution. Lower coverage depth would increase the risk of either failing to make a call or missing one of the two alleles, calling homozygous on a heterozygous genotype.

The proposed technique focused on the main exon sequences for HLA typing by classification of sequencing reads using a comprehensive reference-based mapping strategy. We have shown that a traditional mapping approach using a single sequence as reference is incapable of dealing with the great allelic differences of HLA genes, with a large number of sequencing reads being missed. The comprehensive mapping panel guarantees a full information retrieval from the source data. Classification of sequencing reads to a specific gene first helps avoid the difficulty of *de novo* assembly using all the sequencing reads. HLAreporter also documents the assembled contigs without 100% match to the candidate alleles in the designated database, so it provides a chance of novel allele detection, making full use of the advantages that the NGS technology can bring.

During the revision of this article, Major *et al*. [21] and Bai *et al*. [15] developed two algorithms for HLA typing from NGS data, respectively. The two up-to-date algorithms are both based on an alignment strategy without performing contig assembly, which aim at typing HLA from different NGS data with distinct read lengths, region coverage, and coverage depth. Bai *et al*.’s algorithm PHLAT reported 93% accuracy for WES data at four-digit resolution. We found that a fraction of publicly available WES data they used overlapped with our dataset. Therefore, we checked these overlapped alleles and compared the performance of their method with ours. We found that PHLAT mistyped five alleles out of the 100 alleles, three of which were mistyped at two-digit resolution. HLAreporter outperformed PHLAT with 100% accuracy at four-digit resolution. Notably, these 100 alleles are covered with high quality reads and high depth (Figure S4 in Additional file 1).

Meanwhile, Major *et al*.’s algorithm focused on class I genes and reported 94% accuracy for WES data at four-digit resolution. We tried to replicate their experiments using their WES data but unfortunately their data failed our data quality test (Figure S5 in Additional file 1). This alignment-based algorithm still provides predictions even when the coverage depth is as low as three fold [21]. These data with low coverage depth on the HLA region might only be suitable for an alignment-based approach instead of a *de novo* assembly-based approach such as HLAreporter. We simulated 60 samples based on data used in Major *et al*.’s report (30 WES samples and 30 WGS samples), and correctly predicted all HLA alleles from these samples (see Table S5 in Additional file 1 for details). The simulation suggested that the proposed method also applies to WGS data. For RNAseq data, however, given that the CRP was designed to collect certain short reads with intronic sequences, it might not properly capture the short reads covering exon-exon junctions during mapping; thus, some modification is needed to apply HLAreporter for RNAseq data.

In the current method we propose, only the main exons of HLA genes are examined. These exons determine the amino acid residues of the peptide binding groove that is important for antigen presentation. Yet, there could be a small number of HLA alleles that share identical sequences on the main polymorphic exons while exhibiting polymorphism on other exons, such as between the class I gene C*01:02:01 and C*01:02:11 alleles and between the class II gene DRB1*12:01:01 and DRB1*12:10 alleles. Relatively, these sequences are less important to the binding specificity of the encoded protein [22]. To reach an even higher resolution, further analysis of additional exons would be needed for these alleles.

With efficient HLA calling, analysis such as HLA allele distribution profiles, haplotype prediction, and disease-drug response association studies could be carried out using available NGS data. Indeed, HLA allele frequency estimated here is very consistent with the profile reported from AFND [20]. Interestingly, certain long haplotypes across nearly the entire core MHC region (about 4 Mb) were also observed (Table 3). Although the power is low to accurately estimate the real frequency of these extremely long haplotypes with merely 149 samples, the enrichment of different haplotypes seen in Hong Kong Chinese is consistent with the frequencies of Asian populations in public databases. In summary, the method introduced here is timely and may help us make full use of NGS data and to better connect the alleles in this region with diseases, drug responses, and transplant rejections.

**Table 3:** **HLA long haplotypes observed in our data and their population distribution records in a public database**

**Haplotype HLA A-B-C-DRB1-DQB1**	**Frequency (%)**	**Population in DB** ^**a**^	**DB frequency (%)**
A*33:03-B*58:01-C*03:02-DRB1*03:01-DQB1*02:01	3.36	USA Asian; V; SK; G	2.21; 3.50; 1.90; 0.25
A*02:07-B*46:01-C*01:02-DRB1*14:01-DQB1*05:02	1.34	USA Asian^b^	0.13
A*02:07-B*46:01-C*01:02-DRB1*04:05-DQB1*04:01	1.01	USA Asian	0.11
A*02:07-B*46:01-C*01:02-DRB1*09:01-DQB1*03:03	1.68	USA Asian	1.54; 2.00
A*11:01-B*13:01-C*03:04-DRB1*16:02-DQB1*05:02	0.67	USA Asian; Hispanic	0.18; 0.05
A*11:01-B*15:02-C*08:01-DRB1*15:01-DQB1*06:01	1.68	USA Asian	0.31
A*11:01-B*15:02-C*08:01-DRB1*12:02-DQB1*03:01	3.02	USA Asian; Yunnan^c^	1.41; 1.70-3.40

^a^Full names of the populations in the database are USA Asian pop 2 (USA Asian), Vietnam Hanoi Kinh pop 2 (V), South Korea pop 3 (SK), Germany DKMS-Turkey minority (G), USA Hispanic pop 2 (Hispanic), China Yunan (Yunan). ^b^This population has a relatively large sample size of 1,772 in the database. ^c^Several populations in this Yunnan group with small sample size are not shown.

## Conclusion

This study presents a novel technique for HLA typing from whole exome sequencing data or other NGS data, capable of accurate typing of HLA alleles at high-digit resolution. Accurate HLA typing from NGS data holds much promise for applications in clinical laboratories and biomedical research. Preliminary analysis on both public and local datasets indicates a great potential for broad application of this method.

## Additional file

Additional file 1: Table S1. Exome sequencing data with 601 (top for HapMap data) and 110 known HLA alleles (middle for 1000 Genomes data) and representatives of our 149 NGS samples generated in-house (bottom). **Table S2.** HLA predictions of class I and II genes for 51 HapMap samples (top) and 20 internal samples from Thai population (bottom). **Table S3.** Samples with phase issues. **Table S4.** Average depth of assembled contigs on the exon 2 region which supported the predictions of the HLA-DRB1 gene. **Figure S1.** HLA-DRB1 Prediction of sample PaedA51 is consistent with the PCR-based results. **Figure S2.** Integrative Genomics Viewer image of two samples, PaedA33 and A52 (a) heterozygosity and (b) homozygosity. **Figure S3.** HLA allele frequency in the Hong Kong Chinese population. **Figure S4.** Depth test for sequencing reads of five 1000 Genome samples captured by our comprehensive reference panel. Two thresholds of 50- and 30-fold are indicated in the Figure. **Figure S5.** (a) Depth test for sequencing reads of sample SRR081230 captured by our comprehensive reference panel. Two thresholds of 20- and 30-fold are indicated in the Figure. (b) The number of all informative reads without sequencing error contained in 30 samples. It shows that even though these reads are equally distributed on the targeted exons and are fully captured by the mapping panel, there are still too few of them for proper assembly and use for the detection of the corresponding HLA alleles. Therefore, these samples with poor coverage depth are not suitable for *de novo* assembly-based HLA typing. **Table S5.** HLA predictions of class I genes for 60 WES and WGS samples.

## Abbreviations

aDB: Additional database
AFND: Allele Frequency Net Database
bp: base pair
CRP: comprehensive reference panel
HLA: human leukocyte antigen
mDB: Major database
NGS: next-generation sequencing
SNP: single-nucleotide polymorphism
WES: whole exome sequencing
WGS: whole genome sequencing
