# Supplementary material for: HLAreporter: a tool for HLA typing from next generation sequencing data
Source: Genome Med. 2015 Mar 16;7(1):25. doi: 10.1186/s13073-015-0145-3 (PMC4407542; doi:10.1186/s13073-015-0145-3)
Supplement: Additional file 1: Table S1. — Exome sequencing data with 601 (top for HapMap data) and 110 known HLA alleles (middle for 1000 Genomes data) and representatives of our 149 NGS samples generated in-house (bottom). Table S2. HLA predictions of class I and II genes for 51 HapMap samples (top) and 20 internal samples from Thai population (bottom). Table S3. Samples with phase issues. Table S4. Average depth of assembled contigs on the exon 2 region which supported the predictions of the HLA-DRB1 gene. Figure S1. HLA-DRB1 Prediction of sample PaedA51 is consistent with the PCR-based results. Figure S2. Integrative Genomics Viewer image of two samples, PaedA33 and A52 (a) heterozygosity and (b) homozygosity. Figure S3. HLA allele frequency in the Hong Kong Chinese population. Figure S4. Depth test for sequencing reads of five 1000 Genome samples captured by our comprehensive reference panel. Two thresholds of 50- and 30-fold are indicated in the Figure. Figure S5. (a) Depth test for sequencing reads of sample SRR081230 captured by our comprehensive reference panel. Two thresholds of 20- and 30-fold are indicated in the Figure. (b) The number of all informative reads without sequencing error contained in 30 samples. It shows that even though these reads are equally distributed on the targeted exons and are fully captured by the mapping panel, there are still too few of them for proper assembly and use for the detection of the corresponding HLA alleles. Therefore, these samples with poor coverage depth are not suitable for de novo assembly-based HLA typing. Table S5. HLA predictions of class I genes for 60 WES and WGS samples. [file 13073_2015_145_MOESM1_ESM.pdf]

Table S1. Exome sequencing data with 601 (up table for HapMap data) and 110 known HLA alleles (middle table for 1000 Genomes data) and representatives of our 149 NGS samples generated in-house (down table)

Sample	*Run ID*	*Alternative Run* [a]	*Pedigree*	*Source*	*HLA-A* [b]	*HLA-B*	*HLA-C*	*HLA-DRB1*	*HLA-DQB1*	*HLA-DQA1*
NA18502	SRR764722	SRR764723	Yor004	BCM	2424 7401	1403 5801	0701 0802	1301 0701	0501 0201	0102 0201
NA18505	SRR716648	SRR716649	Yor005	BCM	2601 7401	1503 5301	0202 0701	1503 0301	0602 0201	0102 0501
NA18507	SRR764745	SRR764746	Yor009	BCM	2301 3001	1503 4201	0202 1701	1302 0804	0605 0301	0102 0501
NA18956	SRR766028	-	Japan11	BCM	0201 0201	1501 4002	0303 0304	1502 0901	0601 0303	0103 0301
NA18978	SRR716650	-	Japan26	BCM	0301 3101	4002 4402	0501 1402	1301 0403	0603 0302	0103 0301
NA18994	SRR716431	-	Japan36	BCM	2402 3101	0702 4002	0304 0702	0101 0802	0501 0302	0101 0401
NA18992	SRR716428	-	Japan37	BCM	2402 3303	4403 5201	1202 1403	1302 1502	0604 0601	0102 0103
NA18997	SRR702078	-	Japan38	BCM	2402 2603	1501 3901	0303 0702	0406 -	0302 0303	0301 0301
NA19005	SRR715906	-	Japan42	BCM	0201 3303	5801 6701	0302 0702	1501 1302	0602 0605	0102 0102
NA18508	SRR716637	SRR716638	Yor009	BCM	3303 6802	4201 5301	0401 1701	1303 0302	0201 0402	0201 0401
NA19099	SRR748771	SRR748772	Yor105	BCM	2301 3601	0702 0702	0702 1505	1503 0901	0502 0201	0102 0301
NA18995	SRR764775	-	Japan29	BCM	2402 3303	1507 4403	0303 1403	1302 1101	0604 0301	0102 0501
NA19222	SRR748214	SRR177280	Yor058	BCM	3001 3303	4202 4501	1601 1701	1303 0403	0201 0302	0201 0301
NA18965	SRR764771	SRR764772	Japan20	BCM	2601 3101	4006 5601	0401 0801	0901 0901	0303 0303	0301 0301
NA19137	SRR792560	SRR792542	Yor043	WUGSC	0201 3001	0702 4501	0702 1601	1503 1102	0602 0301	0102 0501
NA19239	SRR792159	SRR792097	Yor117	WUGSC	0201 6802	3501 5201	0401 1601	1301 1201	0501 0301	0103 0501
NA19240	SRR792767	SRR792091	Yor117	WUGSC	3001 6802	3501 5703	0401 1801	1602 1201	0502 0301	0102 0501
NA19238	SRR792121	SRR792165	Yor117	WUGSC	3001 3601	5301 5703	0401 1801	1602 1101	0502 0602	0102 0102
NA18971	SRR077447	SRR078842	Japan30	WUGSC	0206 2402	4002 4002	0304 0702	1401 0901	0503 0303	0101 0301
NA18968	SRR077480	SRR081231	Japan14	WUGSC	1101 3101	4001 5101	0702 1402	15xx 1403	0602 0301	0102 0501
NA18975	SRR078849	SRR081225	Japan23	WUGSC	0201 0206	0702 1501	0401 0702	0101 0406	0501 0302	0101 0301
NA18981	SRR077477	SRR077751	Japan31	WUGSC	0201 3101	5101 5101	1402 1402	0802 1501	0402 0301	0401 0501
NA19152	SRR071135	SRR071167	Yor072	WUGSC	0301 2601	1510 5601	0102 0804	1104 1401	0502 0503	0102 0101
NA19131	SRR070494	SRR070783	Yor101	WUGSC	6602 6802	4201 5301	0401 1701	0302 0804	0402 0301	0401 0501
NA12144	SRR766058	-	1334FF10	BCM	0301 0201	3501 4402	0401 0704	0101 0407	0501 0301	0101 0301
NA07000	SRR766039	-	1340FM10	BCM	0201 6801	4402 4001	0304 0704	0301 1101	0201 0301	0501 0501
NA06985	SRR709972	-	1341MM14	BCM	0301 0201	0702 5701	0702 0602	1501 1501	0602 0602	0102 0102
NA10851	SRR766044	-	1344F01	BCM	2402 0101	4001 0801	0304 0701	0404 0701	0302 0303	0301 0201
NA07357	SRR764689	SRR764690	1345MF12	BCM	2402 0101	3906 0801	0702 0701	0404 0301	0302 0201	0301 0501
NA12044	SRR766060	-	1346FM12	BCM	0201 0101	0702 0702	0702 0702	1301 1501	0603 0602	0103 0102
NA12043	SRR716423	SRR716424	1346FF11	BCM	0206 2601	3501 3801	0401 1203	0101 0404	0501 0302	0101 0301
NA11881	SRR766021	-	1347MF14	BCM	2601 0301	0702 0702	0702 0702	1501 1501	0602 0602	0102 0102
NA11829	SRR710128	-	1350FF10	BCM	0201 0201	4402 1501	0501 0304	0401 0401	0301 0302	0301 0301
NA11832	SRR766003	-	1350MM13	BCM	3201 0201	4002 2705	0202 0704	1301 1501	0603 0602	0103 0102
NA11830	SRR766026	-	1350FM11	BCM	0201 0201	1402 1401	0802 0802	1303 07xx	0301 0201	0501 0201
NA11831	SRR709975	-	1350MF12	BCM	0101 0301	0801 0702	0701 0702	0301 1501	0201 0602	0501 0102
NA11992	SRR701474	-	1362FF13	BCM	0201 0101	3501 0801	0401 0701	0101 0301	0501 0201	0101 0501
NA11995	SRR766010	-	1362MM16	BCM	0101 0101	5701 0801	0602 0701	1301 1501	0603 0602	0102 0103
NA11994	SRR701475	-	1362MF15	BCM	0101 1101	5101 0702	1502 0702	0402 0404	0302 0302	0301 0301
NA12234	SRR716435	-	1375MM12	BCM	3201 1101	4002 4403	1502 1601	0701 1404	0201 0503	0201 0101
NA12156	SRR764691	-	1408MM13	BCM	0101 1101	5101 5001	1502 0602	0407 0701	0301 0201	0301 0201

NA12154	SRR702067	-	1408FF10	BCM	0101 3101	0801 4001	0701 0304	0301 0404	0201 0302	0501 0301
NA12155	SRR702068	-	1408MF12	BCM	0101 0201	0801 4402	0701 0501	0301 0401	0201 0301	0501 0301
NA12005	SRR718067	-	1420MF11	BCM	2902 0201	0702 2705	0702 0102	1501 1501	0602 0602	0102 0102
NA12006	SRR716422	-	1420MM12	BCM	2501 1101	1801 1501	1203 0303	1501 0404	0302 0602	0102 0301
NA12750	SRR794547	SRR794550	1444MF13	WUGSC	3101 0201	1501 0702	0303 0702	1501 0404	0602 0302	0102 0301
NA12814	SRR715914	-	1454MF14	BCM	0301 0201	0702 0702	0702 0702	- -	0501 0602	0101 0102
NA12813	SRR718077	SRR718078	1454FM13	BCM	2902 2402	4403 5701	1601 0602	- -	0303 0201	0201 0201
NA12812	SRR715913	-	1454FF12	BCM	0201 0201	4402 4402	0501 0501	- -	0603 0301	0103 0301
NA12815	SRR716646	-	1454MM15	BCM	0101 2402	0801 5501	0701 0702	- -	0603 0301	0103 0301
NA12873	SRR702070	-	1459FM10	BCM	2402 0301	3905 0702	0702 0702	- -	0602 0602	0102 0102

Sample	*Run ID*	*Capture platform*	*Instrument*	*Read count*	*Exome cov (>10X)*	*HLA-A*	*HLA-B*	*HLA-C*	*HLA-DRB1*	*HLA-DQB1*
HG01756	SRR359102	V2(SureSelect)	GAIIx	190644090	97%	*30:02; *66:01	*18:01; *41:02	*05:01; *17:01	*30:01	*02:01
HG01757	SRR359103	V2(SureSelect)	GAIIx	192645934	97%	*01:01; *02:01	*18:01; *57:01	*07:01	*03:01; *07:01	*02:01; *03:03
NA20313	SRR359108	V2(SureSelect)	GAIIx	195282918	98%	*03:01; *68:02	*35:01; *53:01	*04:01	*04:05; *08:04	*03:01; *03:02
HG01872	SRR359298	V2(SureSelect)	GAIIx	200139823	97%	*11:02; *24:07	*27:04; *39:05	*08:01; *12:02	*08:03; *12:02	*03:01; *06:01
HG01873	SRR359295	V2(SureSelect)	GAIIx	203842382	97%	*02:03; *03:01	*35:03; *55:02	*04:01; *12:03	*08:02; *14:05	*04:02; *05:03
HG01886	SRR360655	V2(SureSelect)	GAIIx	182195662	97%	*30:02; *74:01	*15:03; *57:03	*02:10; *07:01	*11:01; *13:02	*05:02; *06:09
HG01953	SRR360288	V2(SureSelect)	GAIIx	182525408	96%	*02:01; *02:11	*15:04; *35:05	*01:02; *04:01	*04:11; *09:01	*03:02; *03:03
HG01968	SRR360391	V2(SureSelect)	GAIIx	193999096	96%	*02:01; *68:01	*07:02; *40:02	*03:04; *07:02	*01:03; *09:01	*03:03; *05:01
HG02014	SRR360148	V2(SureSelect)	GAIIx	174553282	97%	*02:01; *36:01	*35:01; *40:01	*03:04; *04:01	*01:01; *15:01	*05:01; *06:02
HG02057	SRR359301	V2(SureSelect)	GAIIx	204351266	97%	*02:03; *31:01	*13:01; *48:01	*03:03; *03:04	*11:01; *13:12	*03:01
NA20313	SRR359098	V2(SureSelect)	GAIIx	194492515	98%	*03:01; *68:02	*35:01; *53:01	*04:01	*04:05; *08:04	*03:01; *03:02

Sample	*#Raw reads*	*Target region bp*	*Data size M*	*Read length*	*Mapped to genome*	*Mapped to target*	*Target cov %*	*Target cov>4x*	*Target cov>10x*	*Capture specifi c*
PaedA1	148063241	51543125	13326	90	124515734	89505276	99.44	97.69	94.51	71.22
PaedA2	116956654	51347872	10526	90	95340912	65355832	99.32	97.13	93.21	67.78
PaedA3	157641563	51543125	14188	90	118992693	87820988	99.31	97.37	93.83	73.25
PaedA4	148794674	51543125	13392	90	103762902	76593924	99.14	96.73	92.86	73.28
PaedA5	157641592	51543125	14188	90	118992674	87821023	99.32	97.31	93.81	73.24
PaedA7	165005969	51543125	14850	90	111853314	81085108	99.16	97.06	93.35	71.91
PaedA9	122100356	51347872	10989	90	92961321	68409023	99.05	96.82	93.13	72.86
PaedA11	140192163	51543125	12617	90	85833823	58056769	99.27	97.09	92.85	56.43
PaedA12	148063034	51543125	13325	90	124515598	89505092	99.33	97.62	94.47	71.03
PaedA13	132323957	51543125	11909	90	95084247	69983584	99.08	96.23	92.63	72.06
PaedA14	122865846	51347872	11058	90	102585845	66584585	99.14	96.11	92.93	72.52
PaedA15	122865042	51347872	11058	90	102585374	66584527	99.12	96.08	92.85	72.46
PaedA16	119266612	51543125	10734	90	97101373	71711481	99.16	97.07	93.39	73.39
PaedA17	166212996	51347872	14959	90	112391816	83325596	99.12	96.9	93.22	73.59
PaedA18	167736628	51347872	15096	90	112696002	81226418	99.11	96.91	93.2	71.33
PaedA19	122100890	51347872	10989	90	92961401	68409053	99.11	96.93	93.17	72.99
PaedA20	133218628	51347872	11990	90	110215106	76319230	99.17	96.99	93.24	68.46
PaedA26	140192200	51543125	12617	90	85833955	58056859	99.32	97.16	92.94	56.57
PaedA27	142315946	51543125	12808	90	117887048	65861205	99.57	97.75	93.87	64.55
PaedA28	148794530	51543125	13392	90	103762896	76593848	99.05	96.71	92.83	73.26
PaedA29	165005932	51543125	14851	90	111853392	81085114	99.18	97.06	93.48	71.92

PaedA30	137753804	51347872	12398	90	113655091	78947567	99.32	97.31	93.71	68.68
PaedA31	116956546	51347872	10526	90	95340852	65355780	99.3	97.09	93.15	67.76
PaedA32	148063198	51543125	13326	90	124515662	89505182	99.4	97.66	94.48	71.18
PaedA33	157641478	51543125	14188	90	118992573	87820967	99.27	97.27	93.8	73.22
PaedA34	159652468	51543125	14369	90	115737028	83024750	99.19	97.05	93.39	71.12
PaedA36	125047300	51347872	11254	90	105140414	69903513	99.34	97.21	93.36	65.67
PaedA40	179996380	51347872	16200	90	149952667	84974112	99.52	97.58	93.81	65.66
PaedA51	174975364	51347872	15748	90	142109309	104059718	99.28	97.41	94.18	72.79
PaedA52	179032630	51543125	16113	90	147747939	109983754	99.22	97.42	94.31	74.16

[a]: Samples listed in this column were integrated into their corresponding run (column "Run ID") in an attempt to enhance the data quality for HLA typing. They were released in the same year with their corresponding run (column "Run ID"), with the same read length. [b]: The HLA types of these genes can be found at http://www.inflammgen.org/ and the corresponding publications (de Bakker et al. 2006, Erlich et al. 2011). [c]: Capture specificity is defined as the percentage of uniquely mapped reads aligning to target region.

Table S2. HLA predictions of Class-I and -II genes for 51 HapMap samples (up table) and 20 internal samples from Thai population (down table)

SRR [hmp]	*HLA-A*	*HLA-B*	*HLA-C*	*HLA-DRB1*	*HLA-DQB1*	*HLA-DQA1*
764722		14:03 58:01:01G		**13:01:01G 07:01:01G**	05:01:01G 02:01:01G	**01:02:01G 02:01**
716648	26:01:01G 74:01:01G [p]		**02:02:05 07:01:07** [p]	**15:03:01G 03:01:01G**	**06:02:01G 02:01:01G**	**01:02:01G 05:01:01G**
764745	30:01:01G 23:01:01G [a]			**13:02:01 08:04:01**	*06:09:01 03:01:01G* [a]	*01:02:01G 05:01:01G*
766028				*15:02:01 09:01:02*	**06:01:01G 03:03:02G**	**01:03:01G 03:01:01G**
716650				13:01:01G 04:03:01	**06:03:01G 03:02:01G**	**01:03:01G 03:01:01G**
716431				**01:01:01G 08:02:01**	*03:02:01G 03:02:01G* [e]	01:01:01G 04:01:01G
716428				**13:02:01 15:02:01**	06:04:01G 06:01:01G	**01:02:01G 01:03:01G**
702078				**04:06:01G 09:01:02**		
715906	02:01:09 [a] 33:03:01G [a]			**15:01:01G 13:02:01**	**06:02:01G 06:09:01**	**01:02:01G 01:03:01G**
716637	33:03:01G [a] 68:02:01G [a]			**13:03:01 03:02:01**	02:01:01G 04:02:01	**02:01 04:01:01G**
748771	**23:01:01G 36:01**	**07:02:01G 07:02:01G**		**15:03:01G 09:01:02**	**05:02:01G 02:01:01G**	**01:02:01G 03:01:01G**
764775				*11:01:01G 13:23:02* [l]	*06:04:01G 03:01:01G*	*01:02:01G 05:01:01G*
748214	30:01:01G 33:03:01G [a]	42:02 45:01:01G [p]		13:03:01 04:03:01		*02:01 03:01:01G*
764771				**09:01:02 09:01:02**		*03:01:01G 03:01:01G*
792560	**02:01:01G 30:01:01G**	**07:02:01G 45:01:01G** [p]	**07:02:01G 16:01:01** [p]	**15:03:01G 11:02:01**	**06:02:01G 03:01:01G**	*01:02:01G 05:01:01G*
792159	02:01:01G 68:02:01G [p]		04:01:01G 16:01:01	13:01:01G 12:01:01G	**05:01:01G 03:01:01G**	
792767			04:01:01G 18:01:01G	*16:02:01 12:01:01G*	*05:02:01G 03:01:01G*	
792121	30:01:01G 36:01	53:01:03 57:03:01	**04:01:01G 18:01:01G**	**16:02:01 11:01:02**	**05:02:01G 06:02:01G**	
077447						**01:01:01G 03:01:01G**
077480				**15:01:01G 14:03:01**	**06:02:01G 03:01:01G**	01:02:01G 05:01:01G
078849				**01:01:01G 04:06:01G**	05:01:01G 03:02:01G	**01:01:01G 03:01:01G**
077477				08:02:01 15:01:01G [a]	*04:02:01 03:01:01G*	**04:01:01G 05:01:01G**
071135				11:04:02 14:01:01G	**05:02:01G 05:03:01G**	01:02:01G 01:01:01G

070494				03:02:01 08:04:01	*04:02:01 03:01:01G* [a]	**04:01:01G 05:01:01G**
766058	03:01:01G 02:01:01G [p]	35:01:01G 44:02:01G [p]		**01:01:01G 04:07:01G** [p]	**05:01:01G 03:01:01G**	**01:01:01G 03:01:01G**
766039		44:02:01G [a] 40:01:01G [a]		03:01:01G 11:01:01G	*02:01:01G 03:01:01G*	*05:01:01G 05:01:01G*
709972		07:02:01G 57:01:01G [a]		**15:01:01G 15:01:01G**	**06:02:01G 06:02:01G**	**01:02:01G 01:02:01G**
766044	24:02:01G 01:01:01G [p]	**40:01:01G 08:01:01G** [p]		**04:04:01 07:01:01G**	**03:02:01G 03:03:02G**	
764689				04:04:01 03:01:01G	*02:01:01G 03:02:01G* [a]	
766060		**07:02:01G 07:02:01G**	**07:02:01G 07:02:01G**	**13:01:01G 15:01:01G**	**06:03:01G 06:02:01G**	**01:03:01G 01:02:01G**
716423	02:06:01G 26:01:01G [a]			**01:01:01G 04:04:01** [p]	**05:01:01G 03:02:01G**	01:01:01G 03:01:01G
766021	26:01:01G 03:01:01G [p]	**07:02:01G 07:02:01G**	07:02:01G 07:02:01G	**15:01:01G 15:01:01G**	**06:02:01G 06:02:01G**	*01:02:01G 01:02:01G*
710128	02:01:01G 02:01:01G	15:01:01G 44:34:02 [l]		04:01:01 04:01:01	*03:01:01G 03:02:01G*	03:01:01G 03:01:01G
766003		40:02:01G 27:05:02G [p]	02:02:02G 07:04:01G	**13:01:01G 15:01:01G**	**06:03:01G 06:02:01G**	**01:03:01G 01:02:01G**
766026	02:01:01G 02:01:01G	14:02:01 14:02:01 [l]		13:03:01 07:01:01G	*03:01:01G 02:01:01G*	*02:01 05:01:01G*
709975				**03:01:01G 15:01:01G**	*02:01:01G 06:02:01G*	05:01:01G 01:02:01G
701474				01:01:01G 03:01:01G	05:01:01G 02:01:01G	01:01:01G 05:01:01G
766010				**13:01:01G 15:01:01G**	*06:03:01G 06:02:01G*	01:02:01G 01:03:01G
701475		**51:01:01G 07:02:01G**		**04:02:01 04:04:01**	*03:02:01G 03:02:01G*	03:01:01G 03:01:01G
716435				*07:01:01G 07:01:01G* [e]		02:01 01:01:01G
764691				04:07:01G 07:01:01G	*03:01:01G 02:01:01G*	
702067		**08:01:01G 40:01:01G** [p]		**03:01:01G 04:04:01**	*02:01:01G 03:02:01G*	05:01:01G 03:01:01G
702068		08:01:01G 44:02:01G [a]		*04:01:01 04:01:01* [e]	*02:01:01G 03:01:01G* [a]	05:01:01G 03:01:01G
718067		07:02:01G 27:05:02G	07:02:01G 01:02:01G [p]	**15:01:01G 15:01:01G**	**06:02:01G 06:02:01G**	**01:02:01G 01:02:01G**
716422	25:01:01G 11:01:01G			**15:01:01G 04:04:01**	**03:02:01G 06:02:01G**	01:02:01G 03:01:01G
794547	**31:01:02G 02:01:01G**		03:03:01G 07:02:01G [p]	**15:01:01G 04:04:01**	06:02:01G 03:02:01G	
715914				- -	*06:02:01G 05:01:01G* [a]	01:01:01G 01:02:01G
718077	29:02:01G 24:02:01G		16:01:01 06:02:01G	- -	**03:03:02G 02:01:01G**	**02:01 02:01**
715913				- -	*06:03:01G 03:01:01G* [a]	**01:03:01G 03:01:01G**
716646				- -	*06:03:01G 03:01:01G* [a]	01:03:01G 03:01:01G
702070				- -	06:02:01G 06:02:01G	**01:02:01G 01:02:01G**

ID	*HLAreporter HLA-A* [b]	*Real alleles* [d] *HLA-A*	*Data quality* [c] *-A (>10x >20x >30x >50x)*	*HLAreporter HLA-DRB1*	*Real alleles* [d] *HLA-DRB1*	*Data quality* [c] *-DRB (>10x >20x >30x >50x)*
G3761	02:03:01G; 24 [a]	02:03; 24:02	85%; 52%; 7%; 0%	15:01:01G; 15:02:01	15:01; 15:02	100%; 97%; 94%; 89%
G4045	11:01:01G; 33:03:01G [p]	11:01; 33:03	86%; 55%; 44%; 29%	13:02:01; 14:04	13:02; 14:04	100%; 97%; 91%; 78%
G4047	11:01:01G; 11:01:01G	11:01; 11:01	95%; 76%; 34%; 8%	08:03:02; 14:04	08:03; 14:04	100%; 98%; 90%; 69%
G4049	11:01:01G; 33:03:01G [p]	11:01; 33:03	96%; 73%; 38%; 12%	13:02:01; 15:02:01	13:02; 15:02	100%; 100%; 97%; 94%
G4051	11; 11 [a]	11:01; 11:01	94%; 70%; 41%; 25%	14:04; 15:02:01	14:04; 15:02	99%; 95%; 91%; 79%
G4080	11:01:01G; 26:01:01G	11:01; 26:01	90%; 69%; 33%; 6%	04:05:01; 14:05:01	04:05; 14:05	100%; 97%; 85%; 75%
G4152	02:03:01G; 24:07	02:03; 24:07	92%; 64%; 25%; 0%	12:02:01; 16:02:01	12:02; 16:02	98%; 95%; 91%; 81%
G4153	02; 24 [a]	02:07; 24:07	91%; 64%; 29%; 0%	09:01:02; 12:02:01	09:01; 12:02	99%; 94%; 86%; 68%
G4169	11; 24 [a]	11:01; 24:07	83%; 41%; 30%; 4%	11:01:01G; 14:05:01	11:01; 14:05	100%; 100%; 94%; 76%
G4179	11:01:01G; 26:01:01G	11:01; 26:01	95%; 64%; 34%; 15%	04:05:01; 16:02:01	04:05; 16:02	100%; 97%; 91%; 77%;
G4185	02:01:01G; 02 [a]	02:01; 02:03	91%; 51%; 30%; 10%	14:01:01G; 16:02:01	14:01; 16:02	98%; 95%; 92%; 78%
G4205	01:01:01G; 33:03:01G	01:01; 33:03	85%; 58%; 31%; 0%	09:01:02; 10:01:01	09:01; 10:01	100%; 97%; 87%; 71%

G4207	01:01:01G; 11 [a]	01:01; 11:01	80%; 23%; 0%; 0%	10:01:01; 11:01:01G	10:01; 11:01	100%; 97%; 93%; 74%
G4209	01:01:01G; 02:07:01G	01:01; 02:07	96%; 60%; 31%; 12%	10:01:01; 11:01:01G	10:01; 11:01	100%; 100%; 97%; 83%
G4499	11:01:01G; 11:01:01G	11:01; 11:01	82%; 54%; 36%; 21%	14:05:01; 16:02:01	14:05; 16:02	99%; 92%; 86%; 75%
G4537	02:07:01G; 33 [a]	02:07; 33:03	96%; 61%; 36%; 0%	03:01:01G; 09:01:02	03:01; 09:01	100%; 98%; 90%; 80%
G4545	11; 33 [a]	11:01; 33:03	89%; 50%; 37%; 15%	04:05:01; 16:02:01	04:05; 16:02	100%; 100%; 97%; 85%
G4734	30:01:01G; 02 [a]	02:07; 30:01	75%; 38%; 3%; 0%	07:01:01G; 15:01:01G	07:01; 15:01	100%; 97%; 91%; 77%;
G4736	30:01:01G; 11 [a]	11:01; 30:01	91%; 70%; 34%; 0%	14:05:01; 07:01:01G [a]	07:01; 14:05	96%; 86%; 73%; 54%
G4740	30:01:01G; 30:01:01G	30:01; 30:01	89%; 59%; 18%; 0%	07:01:01G; 07:01:01G	07:01; 07:01	98%; 89%; 79%; 60%

[a]: Additional ambiguity at 4-digit resolution was not shown for this allele; [b]: This HLA gene failed our data quality test (we recommend the value of “>20x” should not be far from 100%. e.g. >=98%) and therefore is not recommended for HLA typing; [c]: “>10x” represents the percentage of locations with coverage depth greater than 10 folds on the targeted exon (>20x, >30x, >50x have the similar definition); [d]: These internal Thai samples were typed using Micro-SSPTM (One lambda, Canoga Park, CA, USA) and LABType® SSO (One Lambda, Canoga Park, CA, USA); [p]: Phase was reported in this sample; [l]: This allele only reached low digit resolution (2-digit); [e]: This allele was not correctly typed at low digit resolution (2-digit); [hmp]: These samples are HapMap samples, whose names are prefixed with “SRR”. For all the HapMap samples, **BOLD** font (e.g. **01:01:01**) represents this gene reaches a data quality of “>10x” = 100% and “>20x” >= 98%; NORMAL font (e.g. 01:01:01) represents this gene only reaches a data quality of “>10x” = 100% and “>20x” >= 90%; *SLASH* font (e.g. *01:01:01*) represents this gene only reaches a data quality of “>10x” >= 95%; Blank entry means this gene failed the pre-set quality threasthold.

Table S3. Samples with phase issue

ID (SRR)	*Prediction*	*Alternative*	*Phase*	*Gap*
359108	A*03:01:01G; A*68:02:01G	A*03:24; *P*	exon2	150 bp
359298	A*11:02:01G; A*24:07	A*11:88; *P* [a]	exon2	130 bp
359295	A*02:03:01G; A*03:01:01G	A*03:89; *P*	inter	intron
360391	A*02:01:01G; A*68:01:02G	A*02:22:01G; *P*	exon3	120 bp
359301	A*02:03:01G; A*31:01:02G	A*02:152; *P*	inter	intron
359098	A*03:01:01G; A*68:02:01G	A*03:24; *P*	exon2	150 bp
359295	B*35:03:01G; B*55:02:01G	B*35:60; *P*	inter [b]	intron
360655	B*15:03:01G; B*57:03:01	B*35:60; *P*	exon3	110 bp
360391	B*07:02:01G; B*40:02:01G	B*07:05:01G; *P*	exon3	120 bp
360148	B*35:01:01G; B*40:01:01G	B*35:63; *P*	inter	intron
359298	C*08:01:01G; C*12:02:01G	C*08:16:01; *P*	exon2 [b]	110 bp
360655	C*02:10; C*07:01:01G	C*02:02:05; *P*	inter	intron
360391	C*03:04:01G; C*07:02:01G	C*03:136; *P*	exon2	150 bp

[a]: Symbol P represents the other allele of this sample, which may or may not be currently documented in IMGT/HLA;
[b]: This sample is with both intra and inter phase. With these gaps on/between exons which are greater than 100 bp, different combinations result in over four different alleles in IMGT/HLA.

Table S4. Average depth of assembled contigs on exon2 region which supported the predictions of HLA-DRB1 gene

Sample	*Allele detected (allele1; allele2)*	*Exon length (bp)*	*Average depth (allele1; allele2)*
PaedA27	*07:01:01G; *10:01:01	270	17; 17
PaedA36	*07:01:01G; *12:02:01	270	18; 13
PaedA40	*09:01:02; *15:01:01G	270	18; 21

Figure S1: HLA-DRB1 Prediction of sample PaedA51 is consistent with the PCR based results.

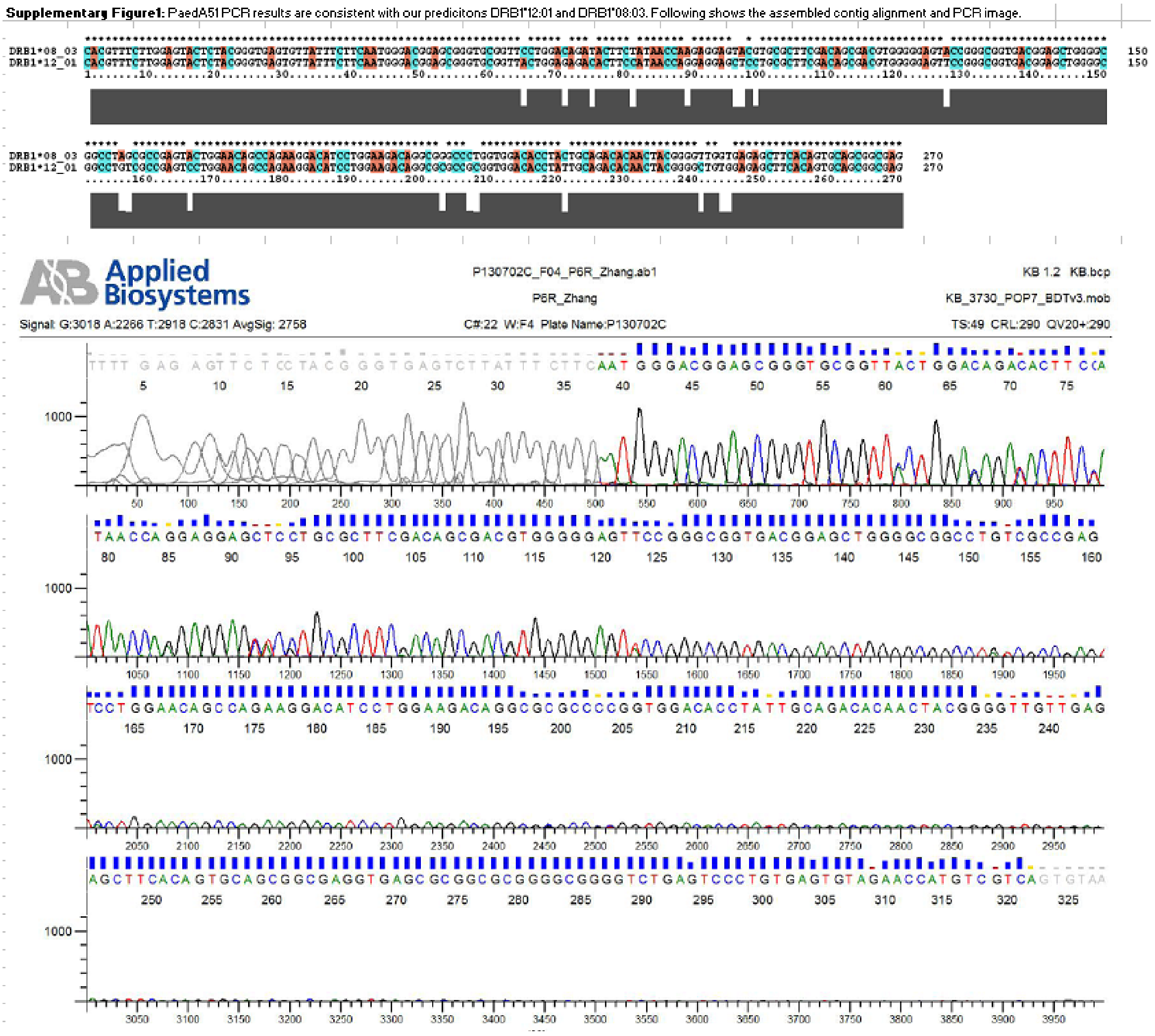

Figure S2: IGV image of two samples PaedA33 and A52 (a) heterozygosity (b) homozygosity.

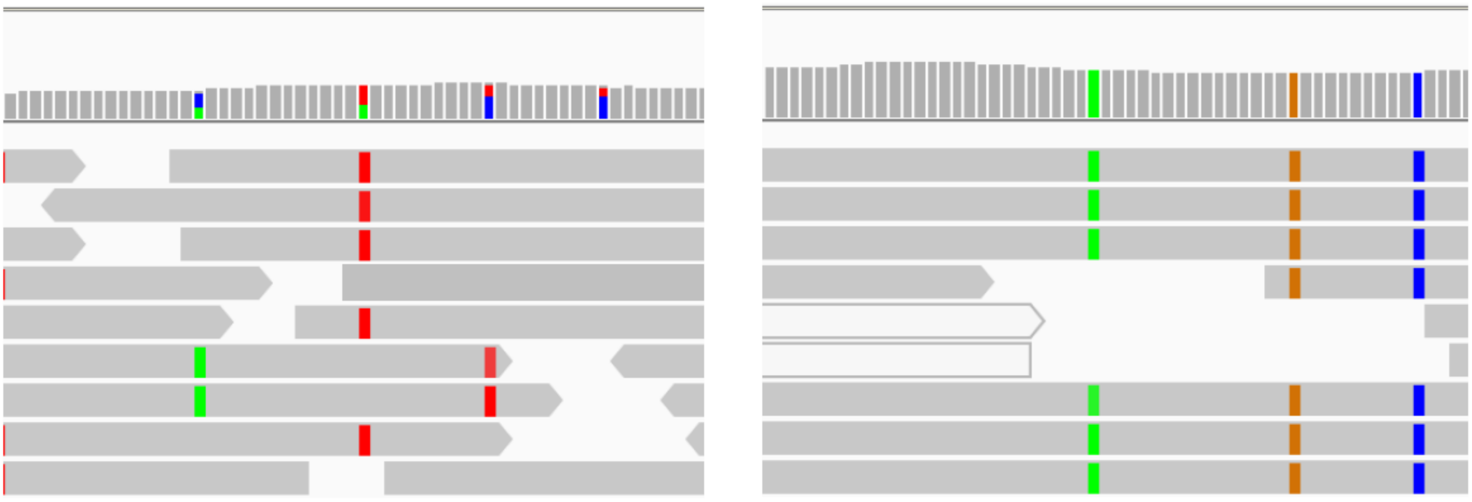

(a) PaedA33 (b) PaedA52

Figure S3: HLA Allele frequency in Hong Kong Chinese population.

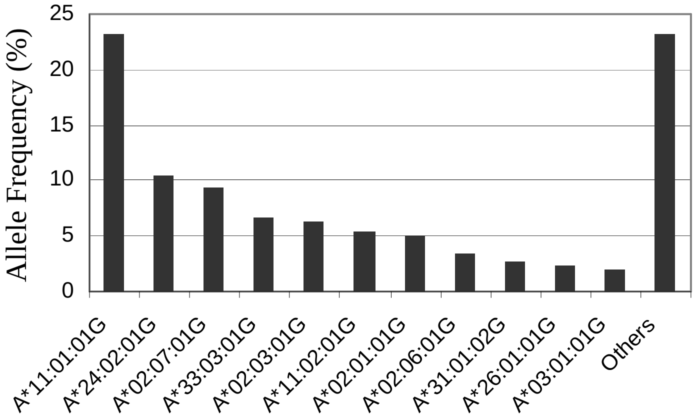

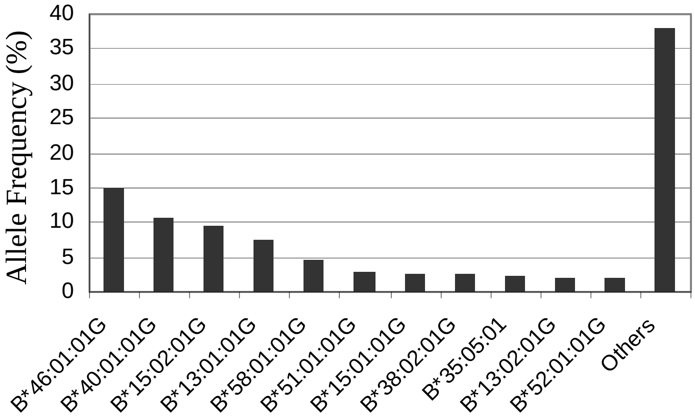

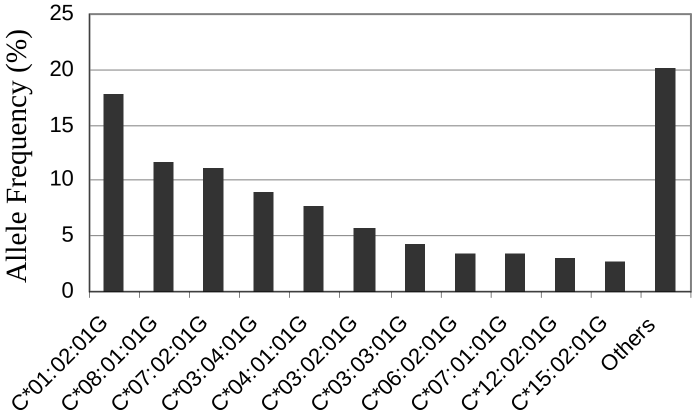

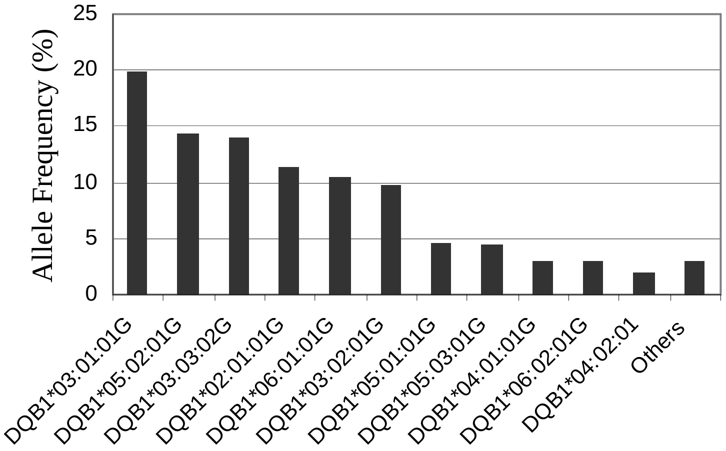

Figure S4: Depth test for sequencing reads of five 1000 Genome samples captured by our comprehensive reference panel. Two thresholds of 50 and 30 folds are indicated in the Figure.

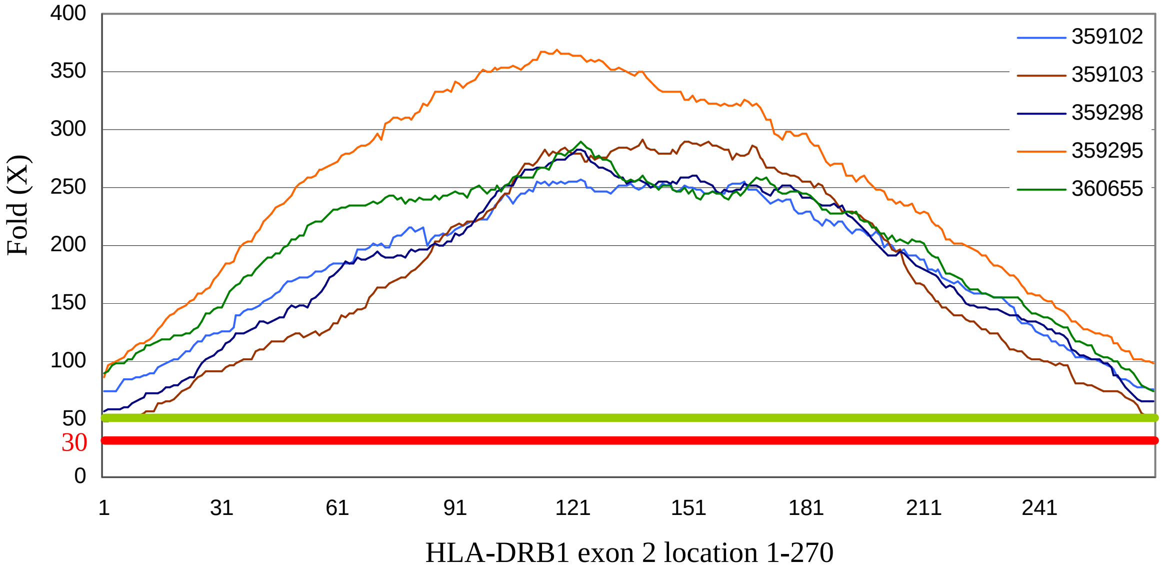

Figure S5: (a) Depth test for sequencing reads of sample SRR081230 captured by our comprehensive reference panel. Two thresholds of 20 and 30 folds are indicated in the Figure. (b) The number of all informative reads without sequencing error contained in 30 samples. It shows that even though these reads are equally distributed on the targeted exons and are fully captured by the mapping panel, there are still too few of them for being properly assembled and used for the detection of the corresponding HLA alleles. Therefore, these samples with poor coverage depth are not suitable for de novo assembly-based HLA typing.

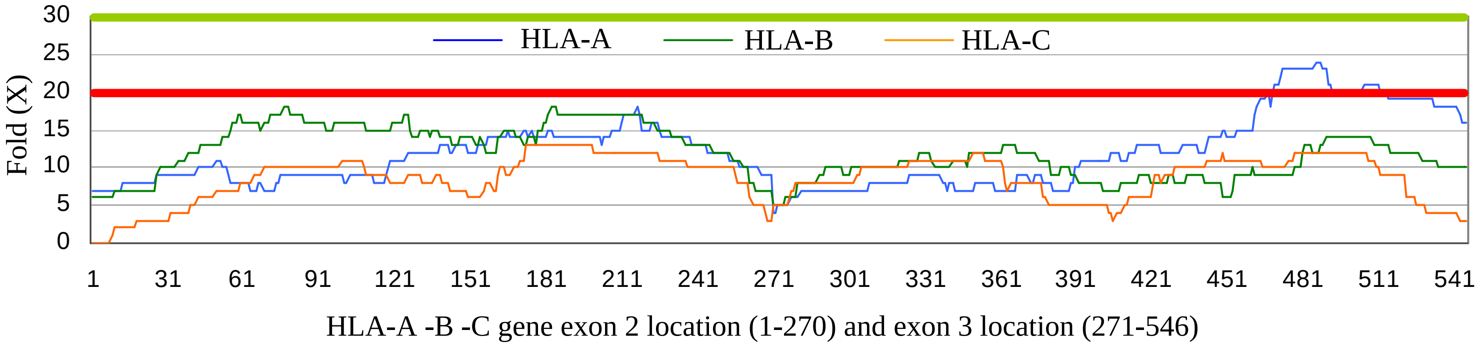

(a)

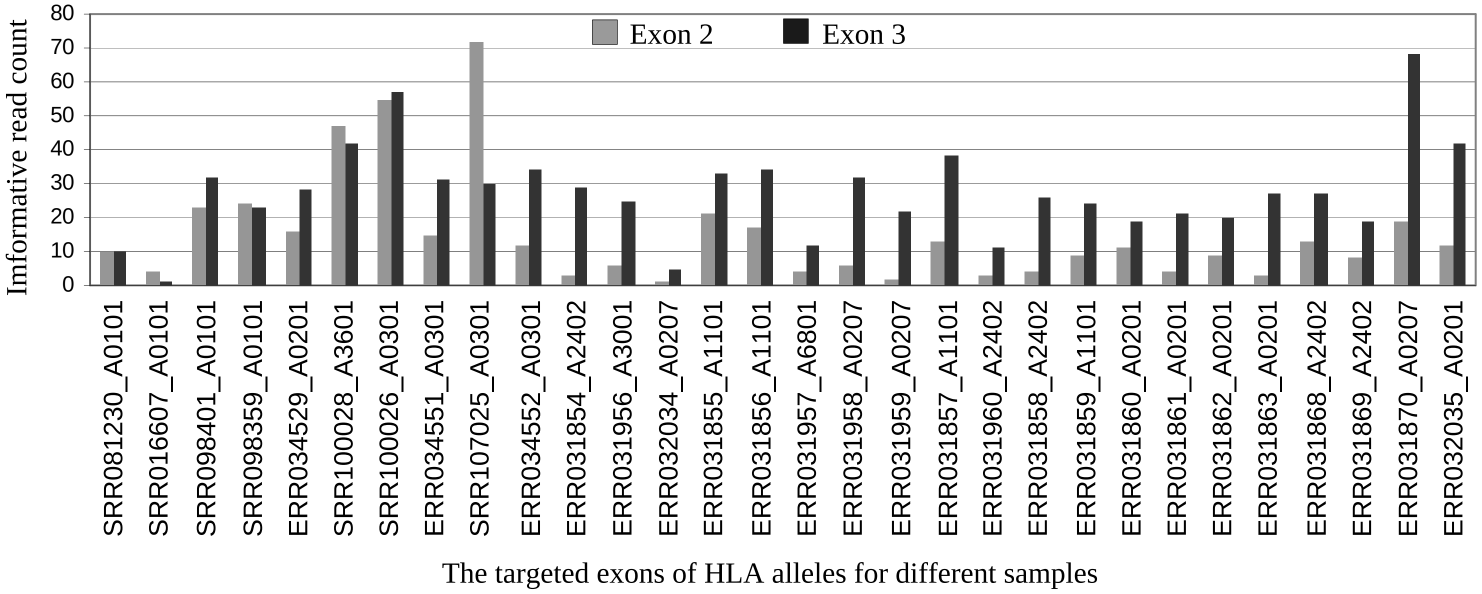

(b)

Table S5. HLA predictions of Class-I genes for 60 WES and WGS samples

ID [a]	HLAreporter HLA-A	Real alleles HLA-A	HLAreporter HLA-B	Real alleles HLA-B	HLAreporter HLA-C	Real alleles HLA-C
SRR081230	*01:01:01G; *03:01:01G [p]	*01:01; *03:01	*07:02:01G; *08:01:01G [p]	*08:01; *07:02	*07:01:01G; *07:02:01G [p]	*07:01; *07:02
SRR016607	*01:01:01G; *11:01:01G [p]	*11:01; *01:01	*08:01:01G; *56:01:01G [p]	*56:01; *08:01	*01:02:01G; *07:01:01G [p]	*01:02; *07:01
SRR098401	*01:01:01G; *11:01:01G [p]	*11:01; *01:01	*08:01:01G; *56:01:01G [p]	*56:01; *08:01	*01:02:01G; *07:01:01G [p]	*01:02; *07:01
SRR098359	*01:01:01G; *24:02:01G [p]	*01:01; *24:02	*07:02:01G; *08:01:01G [p]	*08:01; *07:02	*07:01:01G; *07:02:01G [p]	*07:01; *07:02
ERR034529	*02:01:01G; *11:01:01G	*02:01; *11:01	*15:01:01G; *56:01:01G	*56:01; *15:01	*01:02:01G; *04:01:01G	*01:02; *04:01
SRR100028	*36:01; *36:01	*36:01; *36:01	*15:03:01G; *39:10:01	*15:03; *39:10	*02:10; *12:03:01G [p]	*02:10; *12:03
SRR100026	*03:01:01G; *29:02:01G [p]	*03:01; *29:02	*15:10:01; *53:01:01	*15:10; *53:01	*04:01:01G; *04:01:01G	*04:01; *04:01
ERR034551	*03:01:01G; *74:01:01G [p]	*03:01; *74:01	*49:01:01G; *51:01:01G [p]	*49:01; *51:01	*07:01:01G; *16:01:01 [p]	*07:01; *16:01
SRR107025	*03:01:01G; *36:01	*03:01; *36:01	*15:03:01G; *49:01:01G [p]	*15:03; *49:01	*02:10; *07:01:01G [p]	*02:10; *07:01
ERR034552	*01:01:01G; *03:01:01G [p]	*01:01; *03:01	*07:02:01G; *35:01:01G	*07:02; *35:01	*04:01:01G; *15:05:01G	*04:01; *15:05
ERR031854	*24:02:01G; *33:03:01G [p]	*24:02; *33:03	*40:01:01G; *58:01:01G [p]	*40:01; *58:01	*03:02:01G; *07:02:01G	*03:02; *07:02
ERR031956	*30:01:01G; *68:01:01G	*30:01; *68:01	*13:02:01G; *15:18:01G	*13:02; *15:18	*06:02:01G; *07:04:01G [p]	*06:02; *07:04
ERR032034	*02:07:01G; *11:01:01G	*02:07; *11:01	*38:02:01G; *46:01:01G	*38:02; *46:01	*01:02:01G; *07:02:01G [p]	*01:02; *07:02
ERR031855	*11:01:01G; *33:03:01G [p]	*11:01; *33:03	*46:01:01G; *58:01:01G [p]	*46:01; *58:01	*01:02:01G; *03:02:01G	*01:02; *03:02
ERR031856	*11:01:01G; *24:02:01G [p]	*11:01; *24:02	*40:01:01G; *40:01:01G	*40:01; *40:01	*04:01:01G; *07:02:01G	*04:01; *07:02
ERR031957	*02:03:01G; *68:01:01G [p]	*02:03; *68:01	*15:02:01G; *38:01:01	*15:02; *38:01	*08:01:01G; *12:03:01G [p]	*08:01; *12:03
ERR031958	*02:07:01G; *02:07:01G	*02:07; *02:07	*13:01:01G; *46:01:01G	*13:01; *46:01	*01:02:01G; *03:04:01G	*03:04; *01:02
ERR031959	*02:07:01G; *11:01:01G	*02:07; *11:01	*40:02:01G; *46:01:01G	*40:02; *46:01	*01:02:01G; *15:02:01G	*01:02; *15:02
ERR031857	*02:06:01G; *11:01:01G	*02:06; *11:01	*07:05:01G; *54:01:01G	*07:05; *54:01	*01:02:01G; *07:02:01G [p]	*01:02; *07:02
ERR031960	*02:01:01G; *24:02:01G [p]	*02:01; *24:02	*48:01:01G; *67:01:01 [p]	*48:01; *67:01	*07:02:01G; *08:01:01G	*08:01; *07:02
ERR031858	*24:02:01G; *26:01:01G	*24:02; *26:01	*39:01:01G; *59:01:01G	*39:01; *59:01	*01:02:01G; *07:02:01G [p]	*01:02; *07:02
ERR031859	*11:01:01G; *33:03:01G [p]	*11:01; *33:03	*51:01:01G; *52:01:01G [p]	*51:01; *52:01	*12:02:01G; *14:02:01	*12:02; *14:02
ERR031860	*02:01:01G; *33:03:01G [p]	*02:01; *33:03	*48:01:01G; *51:01:01G	*48:01; *51:01	*08:03:01G; *15:02:01G	*08:03; *15:02
ERR031861	*02:01:01G; *30:01:01G	*02:01; *30:01	*13:01:01G; *13:02:01G	*13:01; *13:02	*03:04:01G; *06:02:01G	*03:04; *06:02
ERR031862	*02:01:01G; *33:03:01G	*02:01; *33:03	*08:01:01G; *35:01:01G [p]	*35:01; *08:01	*03:03:01G; *07:02:01G [p]	*03:03; *07:02
ERR031863	*02:01:01G; *02:06:01G	*02:01; *02:06	*15:01:01G; *15:11:01G	*15:01; *15:11	*03:03:01G; *03:03:01G	*03:03; *03:03
ERR031868	*03:02:01; *24:02:01G [p]	*03:02; *24:02	*40:01:01G; *51:01:01G	*40:01; *51:01	*07:02:01G; *15:02:01G [p]	*07:02; *15:02
ERR031869	*24:02:01G; *31:01:02G [p]	*24:02; *31:01	*15:01:01G; *54:01:01G	*15:01; *54:01	*01:02:01G; *03:03:01G [p]	*01:02; *03:03
ERR031870	*02:07:01G; *02:07:01G	*02:07; *02:07	*46:01:01G; *54:01:01G [p]	*46:01; *54:01	*01:02:01G; *07:02:01G [p]	*01:02; *07:02
ERR032035	*02:01:01G; *02:01:01G	*02:01; *02:01	*15:01:01G; *40:01:01G [p]	*15:01; *40:01	*01:02:01G; *15:02:01G	*01:02; *15:02
ERR050082	*02:01:01G; *03:01:01G [p]	*03:01; *02:01	*07:02:01G; *57:01:01G	*07:02; *57:01	*06:02:01G; *07:02:01G [p]	*07:02; *06:02
ERR068361	*02:01:01G; *03:01:01G [p]	*03:01; *02:01	*07:02:01G; *57:01:01G	*07:02; *57:01	*06:02:01G; *07:02:01G [p]	*07:02; *06:02
SRR400039	*02:01:01G; *03:01:01G [p]	*03:01; *02:01	*07:02:01G; *57:01:01G	*07:02; *57:01	*06:02:01G; *07:02:01G [p]	*07:02; *06:02
SRR400037	*01:01:01G; *03:01:01G [p]	*01:01; *03:01	*07:02:01G; *08:01:01G [p]	*08:01; *07:02	*07:01:01G; *07:02:01G [p]	*07:01; *07:02
SRR385763	*02:01:01G; *32:01:01G [p]	*32:01; *02:01	*27:05:02G; *40:02:01G [p]	*40:02; *27:05	*02:02:02G; *07:04:01G	*02:02; *07:04
SRR393988	*02:01:01G; *02:01:01G	*02:01; *02:01	*27:05:02G; *57:01:01G	*27:05; *57:01	*02:02:02G; *06:02:01G	*02:02; *06:02
SRR393989	*02:01:01G; *02:01:01G	*02:01; *02:01	*27:05:02G; *57:01:01G	*27:05; *57:01	*02:02:02G; *06:02:01G	*02:02; *06:02
ERR050083	*03:01:01G; *26:01:01G [p]	*26:01; *03:01	*07:02:01G; *07:02:01G	*07:02; *07:02	*07:02:01G; *07:02:01G	*07:02; *07:02
ERR068362	*03:01:01G; *26:01:01G [p]	*26:01; *03:01	*07:02:01G; *07:02:01G	*07:02; *07:02	*07:02:01G; *07:02:01G	*07:02; *07:02
SRR385759	*01:01:01G; *02:01:01G	*02:01; *01:01	*08:01:01G; *35:01:01G [p]	*35:01; *08:01	*04:01:01G; *07:01:01G	*04:01; *07:01
SRR385753	*01:01:01G; *11:01:01G [p]	*01:01; *11:01	*07:02:01G; *51:01:01G	*51:01; *07:02	*07:02:01G; *15:02:01G	*15:02; *07:02

SRR385754	*01:01:01G; *01:01:01G	*01:01; *01:01	*08:01:01G; *57:01:01G	*57:01; *08:01	*06:02:01G; *07:01:01G	*06:02; *07:01
SRR385756	*02:01:01G; *02:01:01G	*02:01; *02:01	*08:01:01G; *13:02:01G	*08:01; *13:02	*06:02:01G; *07:01:01G	*07:01; *06:02
ERR162826	*02:01:01G; *24:02:01G	*24:02; *02:01	*07:02:01G; *07:02:01G	*07:02; *07:02	*07:02:01G; *07:02:01G	*07:02; *07:02
SRR385757	*02:01:01G; *24:02:01G	*24:02; *02:01	*07:02:01G; *07:02:01G	*07:02; *07:02	*07:02:01G; *07:02:01G	*07:02; *07:02
SRR385760	*11:01:01G; *25:01:01G	*25:01; *11:01	*15:01:01G; *18:01:01G	*18:01; *15:01	*03:03:01G; *12:03:01G	*12:03; *03:03
ERR162819	*01:01:01G; *02:01:01G	*02:01; *01:01	*07:02:01G; *07:02:01G	*07:02; *07:02	*07:02:01G; *07:02:01G	*07:02; *07:02
SRR385769	*01:01:01G; *31:01:02G [p]	*01:01; *31:01	*08:01:01G; *40:01:01G [p]	*08:01; *40:01	*03:04:01G; *07:01:01G [p]	*07:01; *03:04
SRR385762	*01:01:01G; *02:01:01G	*01:01; *02:01	*08:01:01G; *44:02:01G [p]	*08:01; *44:02	*05:01:01G; *07:01:01G	*07:01; *05:01
SRR393992	*01:01:01G; *11:01:01G [p]	*01:01; *11:01	*50:01:01; *51:01:01G [p]	*51:01; *50:01	*06:06; *15:02:01G [p]	*15:02; *06:06
SRR385773	*23:01:01G; *24:02:01G	*23:01; *24:02	*44:03:01G; *51:01:01G [p]	*44:03; *51:01	*04:01:01G; *14:02:01G	*14:02; *04:01
SRR385775	*02:01:01G; *02:01:01G	*02:01; *02:01	*44:02:01G; *51:01:01G [p]	*51:01; *44:02	*05:01:01G; *14:02:01G [p]	*14:02; *05:01
SRR407508	*02:01:01G; *02:01:01G	*02:01; *02:01	*44:02:01G; *51:01:01G [p]	*51:01; *44:02	*05:01:01G; *14:02:01G [p]	*14:02; *05:01
SRR385776	*24:02:01G; *29:02:01G	*29:02; *24:02	*44:03:01G; *57:01:01G	*44:03; *57:01	*16:01:01; *06:02:01G	*16:01; *06:02
SRR359062	*11:01:01G; *31:01:02G [p]	*11:01; *31:01	*40:01:01G; *51:01:01G	*40:01; *51:01	*07:02:01G; *14:02:01G	*07:02; *14:02
SRR359095	*02:06:01G; *24:02:01G [p]	*02:06; *24:02	*40:02:01G; *40:02:01G	*40:02; *40:02	*03:04:01G; *07:02:01G	*03:04; *07:02
SRR360136	*02:01:01G; *26:01:01G [p]	*02:01; *26:01	*39:01:01G; *40:01:01G	*39:01; *40:01	*07:02:01G; *07:02:01G	*07:02; *07:02
SRR359070	*02:01:01G; *02:06:01G	*02:01; *02:06	*07:02:01G; *15:01:01G	*07:02; *15:01	*04:01:01G; *07:02:01G	*04:01; *07:02
SRR359110	*33:03:01G; *33:03:01G	*33:03; *33:03	*44:03:01G; *44:03:01G	*44:03; *44:03	*14:03:01G; *14:03:01G	*14:03; *14:03
SRR359083	*02:01:01G; *31:01:02G [p]	*02:01; *31:01	*51:01:01G; *51:01:01G	*51:01; *51:01	*14:02:01G; *14:02:01G	*14:02; *14:02

[p]: Phase was reported in this sample.

[a]: These simulated samples are based on 30 WES and 30 WGS samples from the 1000 Genomes Project (Major's paper), respectively. Based on the known HLA-A, -B, -C alleles of these samples, we use the known 6-digit alleles as mapping reference for each sample. We then remove all 0-mismatch sequencing reads towards the mapping reference from each sample. Therefore, all the informative reads that support the corresponding HLA alleles are eliminated, and in the meantime the "noise" reads are retained in the original data. After that, we simulate the paired-end reads from the sequences of their real HLA alleles for each sample (a simulated insert size of 250 bp, a sliding window of 7 bp, and a read length identical with the original sample). The simulation processes are the same between WGS and WES since there is no essential difference in the targeted reads collected for HLA calling between the two technologies. Therefore we have 10 folds of error-free read pairs for each HLA allele. These simulated reads are injected into the two fastq files for each sample to generate the input data to call HLA.
